# Review of patient‐reported outcomes (PROs) and non‐PROs in randomized controlled trials addressing head/neck cancers

**DOI:** 10.1002/cam4.7036

**Published:** 2024-04-22

**Authors:** Michelle Gode, Clovis Mariano Faggion

**Affiliations:** ^1^ Department of Periodontology and Operative Dentistry, Faculty of Dentistry University Hospital Münster Münster Germany

**Keywords:** head/neck cancer, methodological study, methods, randomized controlled trial, systematic review

## Abstract

**Background:**

To assess the frequency of patient‐reported outcomes (PROs) and non‐PROs in randomized controlled trials (RCTs) addressing head/neck cancers.

**Methods:**

We included RCTs about interventions to treat head/neck cancers. PubMed was searched on September 16, 2022 and included studies published during three periods (2000–2002, 2010–2012, and 2020–2022). Data on types of outcomes and instruments to measure them were extracted and organized into PROs and non‐PROs, and temporal trends for reporting outcomes were determined.

**Results:**

There was a reduction in the frequency of non‐PROs (40% to 22%) and an increase in PROs (5% to 19%) over 20 years. The frequency of reporting both non‐PROs and PROs seemed to be stable over the same period (55% to 58%). A great variety of instruments to measure PROs and non‐PROs was identified.

**Conclusions:**

There has been a growth in the types of PROs in more recent years, and they were more frequently reported in RCTs. However, head/neck cancer trials with a combination of PROs and non‐PROs were the most prevalent.

## 1 INTRODUCTION

Patient‐reported outcomes (PROs) are pivotal in clinical research to understand whether therapies have in fact impacted the lives of patients. In contrast, relying only on outcomes from the perspective of the researcher provides a limited view of the potential impact of therapies. For example, Parmar et al.^1^ is a systematic review of compiled evidence on the effect of chemotherapy for treating oral and oropharyngeal cancer. However, it only reports endpoints for survival and locoregional control. Similarly, researchers treating tongue carcinoma with a combination of surgical, radio, and chemical therapies have only reported data on survival and cancer recurrence.^2^ One example of PRO is pain or discomfort after an intervention was applied.^3^ Ideally, clinical studies should present a mixture of PROs and non‐PROs to provide a more comprehensive view of the effects of proposed therapies.

In the head and neck areas, the use of PROs is even more critical in oncology due to the devastating potential side effects of conventional therapies, such as chemotherapy and radiotherapy, which may be long‐lasting or even permanent.^4^ To the best of our knowledge, a comprehensive overview of the types of outcomes in randomized controlled trials (RCTs) of interventions for treating head and neck cancers has not yet been published.

The objective of the present study was to provide a comprehensive mapping of non‐PROs and PROs in RCTs involving the treatment of head and neck neoplasms. We also evaluated whether there was a temporal trend in reporting different types of outcomes.

## 2 METHODS

### 2.1 Research question

The following research questions were used to guide the conduct of the present study: (1) What kinds of non‐PROs and PROs are reported in RCTs involving the treatment of head and neck neoplasms? (2) Are there any temporal trends in the reporting of PROs in RCTs involving the treatment of head and neck neoplasms?

### 2.2 Eligibility criteria

Clinical studies in the form of RCTs of interventions to treat head and neck cancers were included. Furthermore, studies focusing on dealing with or avoiding the side effects of cancer treatments and supportive treatments for cancer were also included. When additional follow‐ups of the original RCTs were found, we selected the article with the longest follow‐up. Other types of study design and RCTs with other aims were excluded. Study protocols, secondary analyses of RCTs, Mendelian randomization designs, studies focusing on other types/areas of cancer, and studies with animals were also excluded. Articles in languages other than English were excluded. Articles reporting information on the same study were excluded.

### 2.3 Definition of non‐PROs and PROs

Non‐patient‐reported outcomes were defined as endpoints that could be measured from the perspective of the researcher; therefore, there was no involvement of patient feedback. In our study all outcomes that were measured from the perspective of the researcher were considered non‐PROs.

Patient‐reported outcomes were defined in our study as information reported by patients, with or without the use of a validated instrument,^5^ that reflected the effects of the received therapies.

We included non‐PROs in order to have a control group to be compared. In this way, the reader will better interpret the prevalence of PROs in comparison to non‐PROs.

### 2.4 Data search and selection

A sample of RCTs was searched in the PubMed database using a predefined strategy (as reported in File S1). The search strategy included medical subject headings (MeSH) terms connected by the Boolean operators “OR” and “AND.” The search was conducted on September 16, 2022 and included articles published during three different periods: 2000–2002, 2010–2012, and 2020–2022.

The title and abstract of each article were first screened to determine inclusion, and if it appeared that an article did not meet the inclusion criteria, it was excluded, and the reason for exclusion was recorded. The full text of each article included from the title/abstract review (the first phase) was then scrutinized, and if the article did not meet the inclusion criteria based on this more in‐depth review, it was excluded, and the reason for exclusion was recorded. Two reviewers (MG and CMF) independently selected a sample of 10% of eligible studies and achieved good agreement (at least 80%), with the remainder selected by one reviewer (MG), as recommended by a validated instrument to assess the methodological quality of systematic reviews.^6^

### 2.5 Data extraction

A spreadsheet (Excel) form was created and used to record the data extracted from outcomes reported in the RCTs. During the development of the extraction form, the two authors met for several sessions to refine the form so that the information could be extracted in the most accurate way possible. Data on the types of outcome measures were extracted and organized into PROs and non‐PROs. We also extracted information on the instruments and approaches used to assess PROs and non‐PROs. To produce the most accurate data extraction process, we transcript what RCT authors reported regarding the name of the instrument, or the form of measurement in a textual form. Furthermore, the following characteristics of the RCTs were extracted: (1) year of publication; (2) country and continent of the first and last authors; (3) type of journal (oral oncology journal, oncology journal, other); (4) name of the journal; (5) type of RCT (parallel, split mouth, other); (6) RCT aim (interventional, other); (7) number of treatment arms; (8) RCT blinding (single‐blind, double‐blind, triple‐blind, not reported/unclear, no blinding); (9) main objective of the RCT (treat cancer, deal with or avoid the side effects of cancer treatment and supportive treatment for cancer); (10) number of centers of the studies (single center, multicenter); (11) protocol registered; (12) name of the registry; (13) ethics committee statement; (14) conflict of interest (COI); (15) sponsorship statement; (16) number of citations in Google Scholar; (17) impact factor [IF; 2022 Journal Impact Factor, Journal Citation Reports (Clarivate, 2023)]; (18) H‐index of the first and last authors; (19) outcome measurement used in the RCT (non‐PROs, PROs, combination, unclear); and (20) primary outcome reported. As with the selection phase, two reviewers (MG and CMF) independently extracted data from a sample of articles, and the results were compared. If there was good agreement (at least 80%), then one reviewer (MG) extracted the remaining data.

### 2.6 Data analysis

Categorical data were reported as frequencies and percentages. When applicable, medians and respective interquartile ranges (IQR) were reported.

## 3 RESULTS

### 3.1 Included articles

**FIGURE 1:**
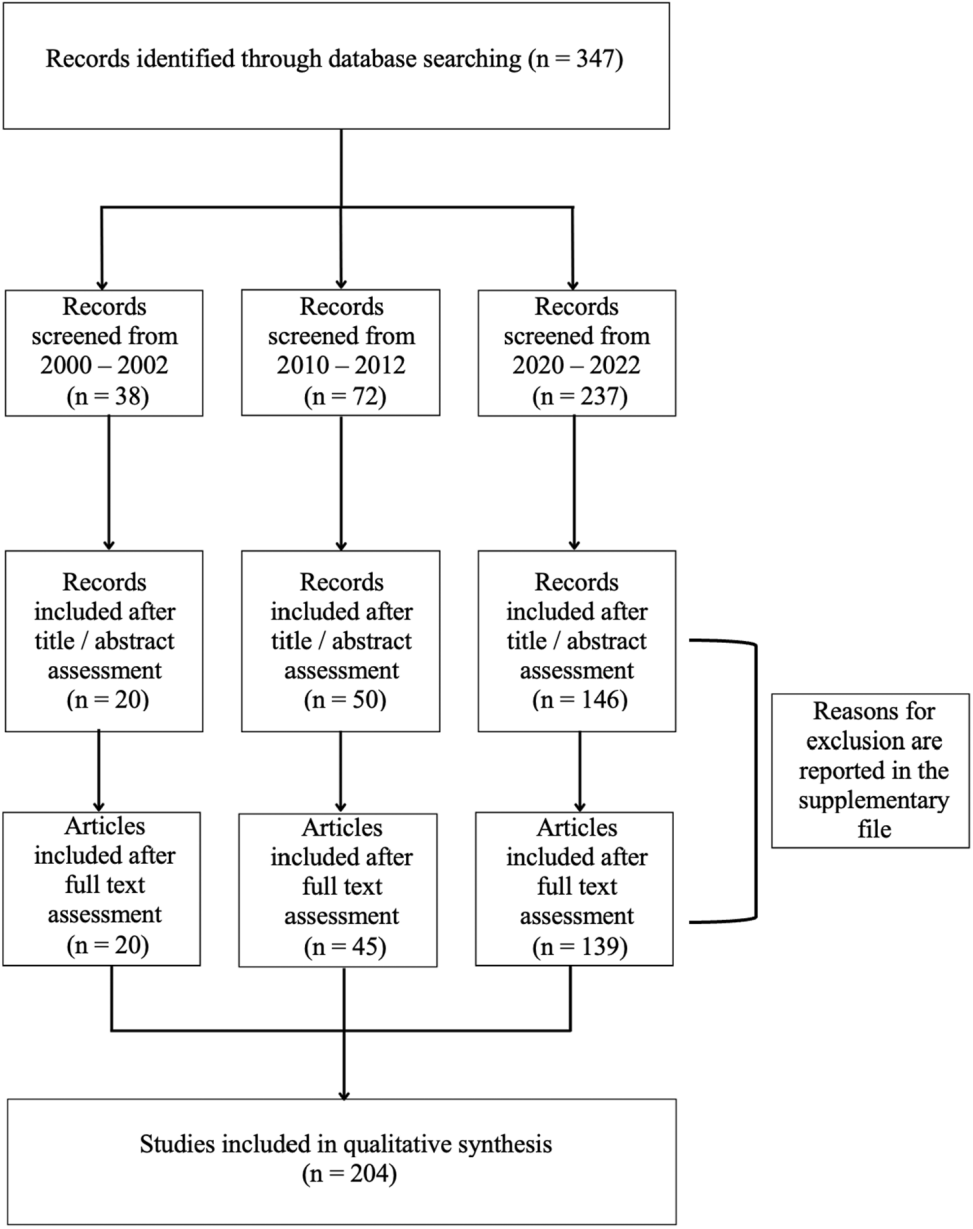
Flowchart of the selection process.

The initial search retrieved 347 documents from the three predefined periods. After the initial review of the titles and abstracts, 131 articles were excluded. After the full text assessment, an additional 12 articles were excluded. The search and selection processes are illustrated in Figure 1. The search strategy is reported in File S1. A full list of included and excluded articles (with reasons for exclusion) is also reported in File S1.

### 3.2 Characteristics of the studies

The majority of RCTs were published in oncology journals (*n* = 119, 58.33%). The most common RCT design was parallel (*n* = 203, 99.51%), and all of the studies had an interventional aim (*n* = 204, 100%). Most of the RCTs had two treatment arms (*n* = 187, 91.67%), and most of their protocols were registered (*n* = 128, 62.75%), predominantly in ClinicalTrials.gov (*n* = 72, 56.25%). The median IF of the journals was 4.856 (IQR = 4.936). Most of the RCTs used a combination of PROs and non‐PROs (*n* = 122, 59.80%) and reported a primary outcome (*n* = 150, 73.53%). All information about the RCT characteristics is reported in Table 1.

**TABLE 1:** Characteristics of the 204 included articles.

Characteristics	Frequency	%
Year of publication		
2020–2022	139	68.14
2010–2012	45	22.06
2000–2002	20	9.80
Continent of the first author		
Africa	0	0.00
Asia	63	30.88
Africa/Asia	4	1.96
Europe/Asia	1	0.49
Australia	4	1.96
Europe	75	36.76
North America	35	17.16
South America	9	4.41
Unclear	13	6.37
Continent of the last author		
Africa	0	0.00
Asia	58	28.43
Africa/Asia	2	0.98
Europe/Asia	1	0.49
Australia	6	2.94
Europe	70	34.31
North America	31	15.20
South America	8	3.92
Unclear	28	13.73
Journal type		
Oral oncology journal	8	3.92
Oncology journal	119	58.33
Other	77	37.75
Journal name		
International Journal of Radiation Oncology Biology Physics	16	7.84
Radiotherapy and Oncology	13	6.37
Journal of Clinical Oncology	9	4.41
Supportive Care in Cancer	9	4.41
European Journal of Cancer (EJC)	8	3.92
Others	149	73.04
RCT type		
Parallel	203	99.51
Split‐mouth	0	0.00
Other	1	0.49
RCT aim		
Interventional	204	100.00
Other	0	0.00
RCT arms		
2	187	91.67
3	14	6.86
4 (and more)	3	1.47
RCT blinding		
Single‐blind	39	19.12
Double‐blind and triple‐blind	62	30.39
Not reported/unclear	50	24.51
No blinding	53	25.98
Main objective of the RCT		
Treat cancer	68	33.33
Treat or avoid the side effect of cancer treatment and supportive treatment for cancer	136	66.67
Number of centers of the study		
Single center	111	54.41
Multicenter	62	30.39
Unclear	31	15.20
Protocol registration?		
Yes	128	62.75
No	76	37.25
Protocol registry name		
ClinicalTrials.gov	72	56.25
Clinical Trials Registry of India (CTRI)	12	9.38
German Clinical Trials Register (DRKS)	5	3.91
ISRCTN Registry	4	3.13
Thai Clinical Trials Registry	3	2.34
Japan Registry of Clinical Trials (jRCT)	3	2.34
Brazilian Clinical Trials Registry (REBEC)	3	2.34
Iranian Registry of Clinical Trials (IRCT)	3	2.34
Netherlands Trial Registry (NTR)	3	2.34
Australian New Zealand Clinical Trials Registry (ACTRN)	3	2.34
Other	17	13.28
Ethics committee reported		
Yes	179	87.75
No	25	12.25
Conflict of interest statement reported		
Yes	155	75.98
No	49	24.02
Statement on funding reported		
Yes	155	75.98
No	49	24.02
Number of citations (Google Scholar)		
Median (IQR)	11 (47.75)	
Impact factor (IF)		
Median (IQR)	4.856 (4.936)	
H index of the first author		
Median (IQR)	9 (18)	
H index of the last author		
Median (IQR)	21 (30.5)	
Outcome measure used in the RCT		
Surrogate (non‐PRO)	50	24.51
Patient‐reported outcome (PRO)	31	15.20
Combination of both	122	59.80
Unclear	1	0.49
Primary outcome reported?		
Yes	150	73.53
No	54	26.47

Abbreviation: ISRCTN, International Standard Randomized Controlled Trial Number.

Almost all the RCTs published in 2020–2022 had an ethics committee reported (*n* = 136, 97.84%) (Table S1). A majority of the RCTs published in 2010–2012 did not have a protocol registered (*n* = 30, 66.67%), but a larger majority had an ethics committee reported (*n* = 34, 75.56%) (Table S2). None of the RCTs published in 2000–2002 had a protocol registered (*n* = 20, 100%) (Table S3). Moreover, just under half of the RCTs published in 2000–2002 had an ethics committee reported (*n* = 9, 45%). All information about the RCT characteristics for the different periods is reported in Tables S1–S3.

### 3.3 Types of non‐PROs and PROs

The most frequent non‐PROs of all periods were “overall survival” (*n* = 72, 35.29%), “adverse events” (*n* = 33, 16.18%), and “progression‐free survival” (*n* = 31, 15.20%) (Table 2). The most frequent PROs of all periods were “pain” (*n* = 66, 32.35%) and “quality of life” (*n* = 56, 27.45%) (Table 3). The RCTs published in 2020–2022 reported “overall survival” (*n* = 43, 30.94%) as the most frequent non‐PRO (Table S4). The RCTs published in 2010–2012 reported “overall survival” (*n* = 21, 46.67%) as the most frequent non‐PRO (Table S5). The RCTs published in 2000–2002 reported “toxicity” and “overall survival” (*n* = 8, 40%) as the most frequent non‐PROs (Table S6). The RCTs published in 2020–2022 reported “pain” (*n* = 47, 33.81%) as the most frequent PRO (Table S7). The RCTs published in 2010–2012 reported “pain” (*n* = 12, 26.67%) as the most frequent PRO (Table S8). The RCTs published in 2000–2002 reported “pain” (*n* = 7, 35%) as the most frequent PRO (Table S9).

**TABLE 2:** The 20 most prevalent non‐patient‐reported outcomes (non‐PROs) in the 204 included articles.

Outcome	*N*	(%)
1. Overall survival	72	35.29
2. Adverse events, adverse effects, serious adverse events	33	16.18
3. Progression‐free survival	31	15.20
4. Objective response rate, overall response rate, response rate, tumor response	29	14.22
5. Oral mucositis, mucositis, severe oral mucositis, radiation induced oral mucositis	26	12.75
6. Toxicity, acute and late toxicity	26	12.75
7. Disease specific survival, disease free survival	21	10.29
8. Amount of saliva, sticky saliva, saliva production, salivary flow	14	6.86
9. Locoregional control	14	6.86
10. Xerostomia	12	5.88
11. Blood loss, blood pressure, blood tests	12	5.88
12. Morbidity	11	5.39
13. Distant metastasis, metastases	10	4.90
14. Dysphagia	9	4.41
15. Safety of treatments	8	3.92
16. Analgesia, opiod use	5	2.45
17. Hospitalization, hospital stay, days in the hospital	5	2.45
18. Death	5	2.45
19. Healing time	3	1.47
20. Oral health	3	1.47

**TABLE 3:** The 20 most prevalent patient‐reported outcomes (PROs) in the 204 included articles.

Outcome	*N*	(%)
1. Pain	66	32.35
2. Quality of life	56	27.45
3. Adverse events, adverse effects, serious adverse events	24	11.76
4. Weight, weight loss, weight changes, body weight (BMI)	23	11.27
5. Xerostomia, dry mouth	23	11.27
6. Swallowing, swallowing difficulties, swallowing problems	18	8.82
7. Nausea	17	8.33
8. Vomiting	12	5.88
9. Toxicity	12	5.88
10. Taste, taste loss, taste changes	10	4.90
11. Amount of saliva, sticky saliva, saliva production, salivary flow	9	4.41
12. Depression	9	4.41
13. Fatigue	8	3.92
14. Patients´ satisfaction	8	3.92
15. Compliance, treatment compliance	7	3.43
16. Social eating, social functioning	6	2.94
17. Patients´ adherence to treatment	6	2.94
18. Mouth opening	6	2.94
19. Symptom burden	4	1.96
20. Appetite loss	3	1.47

### 3.4 Types of instruments for assessing non‐PROs and PROs

For the most frequent non‐PRO (overall survival), the Kaplan–Meier Method was usually applied (Table 4). For the most frequent PRO (pain), the Visual Analog Scale (VAS), the Numerical Rating Scale (NRS), and the McGill Pain Questionnaire were used (Table 5). The Common Terminology Criteria for Adverse Events (CTCAE) was applied to assess the non‐PRO “adverse events.” Across the different periods, there was a growth of different versions of CTCAE. The detailed information on types of instruments used to assess non‐PROs and PROs are reported in Tables S10–S15.

**TABLE 4:** Types of instruments utilized for non‐patient‐reported outcomes (non‐PROs) of all 204 articles.

Outcomes	Measured by
1. Overall survival	Overall survival rate (by Kaplan–Meier method); date of diagnosis until the date of death or the last follow‐up
2. Adverse events, adverse effects, serious adverse events	The National Cancer Institute Common Toxicity Criteria for Adverse Events Version 5.0, using different CTACE versions (3.0/4.0/4.02/5.0)
3. Progression‐free survival	Kaplan–Meier Method
4. Objective response rate, overall response rate, response rate, tumor response	Time to objective response, overall response rate (ORR)
5. Oral mucositis, mucositis, severe oral mucositis, radiation induced oral mucositis	CTCAE V4.0; the Oral Mucosal Assessment Scale; ulcer score; Mucositis grade by WHO
6. Toxicity, acute and late toxicity	CTC 3.0; by blood tests, by EORTC/RTOG scale, European Organization for Research and Treatment of Cancer (EORTC), Radiation Oncology Group (RTOG)
7. Disease specific survival, disease free survival	Time; disease‐free actuarial survival rates
8. Amount of saliva, sticky saliva, saliva production, salivary flow	UWSFR (unstimulated whole salivary flow rates); SSFR (stimulated salivary flow rates); salivary gland scintigraphy; saliva production
9. Locoregional control	Locoregional control rates; Kaplan–Meier Method
10. Xerostomia	Clinical symptoms of xerostomia by RIXVAS (radiation‐induced xerostomia Visual Analog Scale), the objective grade by two separate observers; RTOG (radiation oncology group grades); The National Cancer Institute Common Toxicity Criteria for Adverse Events Version 5.0 [CTCAE v5.0] for the level of xerostomia
11. Blood loss, blood pressure, blood tests	Intraoperative blood loss, blood tests
12. Morbidity	Early and late treatment‐related morbidity
13. Distant metastasis, metastases	Regional metastasis, defined as nodal positive either identified from elective neck dissection at the time of surgery or from postsurgery follow‐up, or distant metastasis
14. Dysphagia	MD Anderson Dysphagia Inventory (MDADI) questionnaire
15. Safety of treatments	Bidirectional measurements, obtained from electronic patient records
16. Analgesia, opiod use	Duration and dose of use of opiate analgesia
17. Hospitalization, hospital stay, days in the hospital	Time
18. Death	Death rate
19. Healing time	Time
20. Oral health	Oral health status, measured by a nurse

**TABLE 5:** Types of instruments utilized for patient‐reported outcomes (PROs) of all 204 articles.

Outcome	Measured by
1. Pain	VAS (Visual Analog Scale); NRS (Numerical Rating Scale); McGill Pain Questionnaire
2. Quality of life	QLQ‐C30 (Quality of Life Questionnaire includes 30 items grouped into five quality of life categories: physical, social, emotional, cognitive, and role performance); Core Module (QLQ‐C30) and Head and Neck Module (QLQ‐H&N35), MDADI, MDASI‐HN (MD Anderson Dysphagia Inventory and Symptom Inventory for head and neck cancer); (EORTC QLQ‐C30); (EORTC QLQ‐HN35, EORTC QLQ‐LC13); (EORTC QLQ‐PATSAT)
3. Adverse events, adverse effects, serious adverse events	Questionnaire
4. Weight, weight loss, weight changes, body weight (BMI)	Questionnaire, weight measurement
5. Xerostomia, dry mouth	TESS (Treatment‐Emergent Symptom Scale); VAS (Visual Analog Scale); Xerostomia Evaluation (XQ‐I) questionnaire and MD Anderson Dysphagia Inventory (MDADI) questionnaire: (using a self‐report instrument, XQ)
6. Swallowing, swallowing difficulties, swallowing problems	Questionnaire
7. Nausea	PAS (penetration and aspiration scale); video fluoroscopic evaluation of swallowing (VFSS); Swallowing Performance Scale (SPS), Swallowing function (by FOIS and PSS‐H&N): FOIS (Functional Oral Intake Scale); PSS‐H&N (Performance Status Scale for Head and Neck Cancer Patients); PBQ (patient benefit questionnaire)
8. Vomiting	Questionnaire
9. Toxicity	European Organization for Research and Treatment of Cancer (EORTC); questionnaire
10. Taste, taste loss, taste changes	Questionnaire
11. Amount of saliva, sticky saliva, saliva production, salivary flow	UWSFR (unstimulated whole salivary flow rates); SSFR (stimulated salivary flow rates)
12. Depression	PROMIS (Patient‐Reported Outcomes Measure Information System); (by EQ‐5D‐3L and EORTC QLU‐C10D), Beck's Depression Inventory; the Hospital Anxiety and Depression Scale (HADS)
13. Fatigue	BFI (Brief Fatigue Inventory), global fatigue score
14. Patients´ satisfaction	The Patient Concerns Inventory (PCI); preoperative and postoperative questionnaires
15. Compliance, treatment compliance	Questionnaire
16. Social eating, social functioning	EORTC QLQ‐H&N35
17. Patients´ adherence to treatment	Participants rated the extent to which they followed their treatment plan and followed their doctor's instructions
18. Mouth opening	MMO (maximum mouth opening)
19. Symptom burden	H&N35; ESAS (Edmonton Symptom Assessment System)
20. Appetite loss	Questionnaire

### 3.5 Temporal trends

**FIGURE 2:**
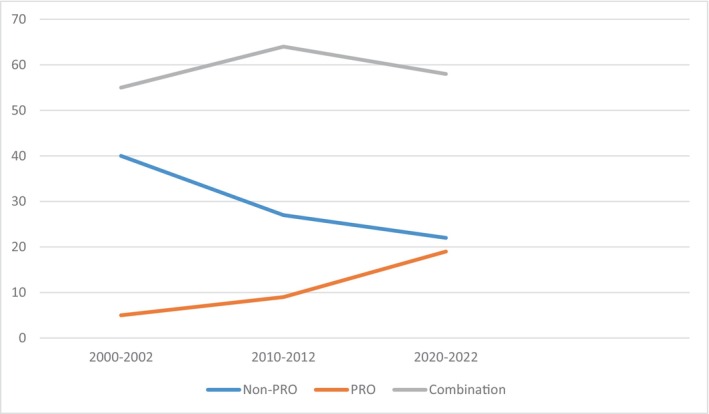
Temporal trend of the reporting of patient‐reported outcomes (PROs) and non‐PROs is randomized controlled trials of interventions for head and neck cancers.

There has been an increase in the reporting of PROs over the last two decades, and a decrease of non‐PROs during the same period. The frequency of RCTs reporting a combination of PROs and non‐PROs was stable over the years. Figure 2 and Table S16 (in File S1) show the temporal trends in the reporting of the outcomes in the 204 RCTs.

## 4 DISCUSSION

### 4.1 Key findings

This study assessed the types of outcomes used in RCTs on oncologic interventions in the head and neck areas. We found a great variety of outcomes and instruments to measure them in RCTs addressing interventions for the treatment of cancer in the head and neck area. We also found a substantial increase in different types of both non‐PROs and PROs in more recent oncologic trials. PROs seemed to be reported more in recent years, although a great proportion of trials reported a combination of PROs and non‐PROs in all the three time periods of the analysis.

### 4.2 Interpretation of the findings

The great variety of types of outcomes in the head and neck interventions for cancer treatment may indicate that the research community intended to cover all aspects of research and understand the impact of these interventions and the corresponding research on patients´ health. In recent years, more attention has been paid to trying to understand how the different therapeutic approaches affect the individual life of a patient with cancer. Probably everyone will agree that the most important outcome is the survival of a patient with cancer. However, other important outcomes, such as quality of life, also play an important role for patients suffering from cancer and the clinicians working with them.^7^

Attention to the effects of cancer therapies is incredibly important in the head and neck area. Conventional therapies, such as chemotherapy and radiotherapy, can cause strong side effects that are temporary or permanent. For example, chemotherapy that may be used to treat lymphoma in the Waldeyer's ring^8^ might impose temporary limitations on the patient due to mucositis, which makes deglutition very difficult. Radiotherapy in this area can also have a similar effect. However, depending on the level of radiation that is applied to the head and neck area, the effects can be more intense and long‐lasting. Trismus^9^ and osteoradionecrosis^10^ are conditions that can be severe and strongly affect a patient's quality of life.

The great variety of PROs might be related to the large number of instruments used to assess PROs. For example, a recent systematic review^11^ identified 116 different instruments to assess health‐related quality‐of‐life outcomes after interventions for treating head and neck cancer. We also found a great variety of instruments used to measure non‐PROs and PROs in the present sample of RCTs. Many of these instruments are validated or in process of validation. However, a great number of types of outcomes and instruments to measure them also make comparison among RCTs more difficult, for example, in the case of a meta‐analysis is conducted. It would be important to determine the priorities for applying PROs and non‐PROs in head and neck cancer research. By classifying the most important outcomes, it is possible to increase efficiency in research with the possibility of reproducibility of findings^12^ by another research team using a similar methodology. For example, some trials have reported the effect of therapies on the saliva of patients with cancer in the form of “amount of saliva,” “consistence of saliva,” and “flow of saliva.” Although this kind of approach can provide an overall comprehensive view of the effects of therapies, it might hinder the reproducibility of findings when new trials are planned for the same research question. Hence, determining the most important outcomes might help to increase efficiency in research and reduce waste with regard to resources.^13^

An interesting finding in our study is the attention given to the research questions in RCTs from different periods. We noticed an increase in RCTs dealing with side effects and the supportive treatment of patients with head and neck cancers during the last 20 years (*p* = 0.025, chi‐squared test with Yates correction) (Tables S1–S3; File S1). This finding may mean that researchers are becoming more interested in the management of potential side effects that would limit the quality of life of patients with cancer. More recent trials also reported a greater number of protocol registrations in public registries, as well as ethics committee permissions to conduct the trials. One can argue that these findings can be explained by a greater awareness in society of the importance of robust methodological principles and ethical aspects regarding the treatment of patients with head and neck cancer.

It is important to reduce the gap between the assessment of PROs in oncological clinical research and the assessment of PROs in clinical practice. Some evidence suggests that only a few health practitioners in clinical oncology use PROs in routine cancer care.^14^ In other words, caregivers dealing with patients with cancer do not effectively listen to their patients. In the current study, the same outcome could be considered both non‐PRO and PRO. The difference would be related to the perspective of who would provide feedback about the effect of the treatment, researcher, or patient. For example, xerostomia could be assessed by a standardized method to assess the degree of dry mouth (researcher's perspective) as well as a more subjective view on the impression of the patients about the dryness of their mouth. Probably, the most important outcome in this situation would be PRO because it might better reflect the effect of the therapy than that measured by the researcher. Therefore, it is pivotal that caregivers pay attention to what patients report about the effects of the applied therapies.

### 4.3 Limitations and strengths

The present study has limitations. There was a difference in the number of trials included in each period examined, which makes a comparison challenging. However, this output was directly related to the predefined eligibility criteria, and the differences can be explained by an increase in the published number of RCTs in more recent years.

This study also has strengths. To the best of our knowledge, this is the first study to assess PROs and non‐PROs reported in RCTs related to the therapy of head and neck cancers. We also included a sizable number of RCTs that might be representative of the current approaches used by researchers to understand the effect of therapies on patients with head and neck cancers.

## 5 CONCLUSIONS

This study reviewed important information on non‐PROs and PROs within the study of the head and neck. It showed that there has been an increase in the reporting of PROs in more recent RCTs addressing head and neck cancers. The present findings provide an overall view of the types of outcomes in head and neck oncology and can support further discussion on the importance of these outcomes in planning future RCTs in the field.

## AUTHOR CONTRIBUTIONS

**Michelle Gode:** Conceptualization (equal); data curation (lead); writing – original draft (equal); writing – review and editing (equal). **Clovis Mariano Faggion Jr:** Conceptualization (equal); project administration (supporting); supervision (lead); writing – original draft (equal); writing – review and editing (equal).

## CONFLICT OF INTEREST STATEMENT

The authors have no conflict of interest to declare.

## Supporting information


Data S1.

## Data Availability

The data that support the findings of this study are available on request from the corresponding author.

## References

[1] Parmar A , Macluskey M , Mc Goldrick N , et al. Interventions for the treatment of oral cavity and oropharyngeal cancer: chemotherapy. Cochrane Database Syst Rev. 2021;2021:CD006386. doi:10.1002/14651858.CD006386.pub4 PMC8687638 34929047

[2] Kokemueller H , Rana M , Rublack J , et al. The Hannover experience: surgical treatment of tongue cancer – a clinical retrospective evaluation over a 30 years period. Head Neck Oncol. 2011;3:27. doi:10.1186/1758-3284-3-27 21600000 PMC3123311

[3] Reuter‐Selbach MJ , Su N , Faggion CM Jr . assessment of the frequency of reporting dental patient‐reported outcomes (dPROs) in a sample of randomized controlled trials on root coverage procedures. J Evid Based Dent Pract. 2023;23:101793. doi:10.1016/j.jebdp.2022.101793 36707163

[4] MD Anderson Head and Neck Cancer Symptom Working Group . Self‐reported oral morbidities in long‐term oropharyngeal cancer survivors: a cross‐sectional survey of 906 survivors. Oral Oncol. 2018;84:88‐94. doi:10.1016/j.oraloncology.2018.07.006 30115482 PMC11349715

[5] Jokstad A . Patient‐reported outcomes (PROs) versus patient‐reported outcome measures (PROMs)‐is there a difference? Clinical and Experimental Dental Research. 2018;4:61‐62. doi:10.1002/cre2.112 29955388 PMC6010775

[6] Shea BJ , Reeves BC , Wells G , et al. AMSTAR 2: a critical appraisal tool for systematic reviews that include randomised or non‐randomised studies of healthcare interventions, or both. BMJ (Clinical Research Ed). 2017;358:j4008. doi:10.1136/bmj.j4008 PMC5833365 28935701

[7] Williams CP , Miller‐Sonet E , Nipp RD , Kamal AH , Love S , Rocque GB . Importance of quality‐of‐life priorities and preferences surrounding treatment decision making in patients with cancer and oncology clinicians. Cancer. 2020;126:3534‐3541. doi:10.1002/cncr.32961 32426870

[8] Shin H‐J , Suh C , Il LS , et al. Clinical characteristics and outcomes of Waldeyer's ring lymphoma: nation‐wide study in Korea. Blood. 2013;122:4332. doi:10.1182/blood.V122.21.4332.4332

[9] Abboud WA , Hassin‐Baer S , Alon EE , et al. Restricted mouth opening in head and neck cancer: etiology, prevention, and treatment. JCO Oncology Practice. 2020;16:643‐653. doi:10.1200/OP.20.00266 33049177

[10] El‐Rabbany M , Duchnay M , Raziee HR , et al. Interventions for preventing osteoradionecrosis of the jaws in adults receiving head and neck radiotherapy. Cochrane Database Syst Rev. 2019;2019:CD011559. doi:10.1002/14651858.CD011559.pub2 31745986 PMC6953365

[11] Cao AC , Lu JS , Hobday SB , et al. Patient‐reported outcomes in head and neck cancer: a systematic review of clinical trials. Int J Radiat Oncol Biol Phys. 2022;112:e60. doi:10.1016/j.ijrobp.2021.12.138

[12] Wallach JD , Boyack KW , Ioannidis JPA . Reproducible research practices, transparency, and open access data in the biomedical literature, 2015‐2017. PLoS Biol. 2018;16:e2006930. doi:10.1371/journal.pbio.2006930 30457984 PMC6245499

[13] Chapman SJ , Aldaffaa M , Downey CL , Jayne DG . Research waste in surgical randomized controlled trials. Br J Surg. 2019;106:1464‐1471. doi:10.1002/bjs.11266 31393612

[14] Cheung YT , Chan A , Charalambous A , et al. The use of patient‐reported outcomes in routine cancer care: preliminary insights from a multinational scoping survey of oncology practitioners. Support Care Cancer. 2022;30:1427‐1439. doi:10.1007/s00520-021-06545-7 34524527 PMC8440726

