# Supplementary material for: Review of patient‐reported outcomes (PROs) and non‐PROs in randomized controlled trials addressing head/neck cancers
Source: Cancer Med. 2024 Apr 22;13(8):e7036. doi: 10.1002/cam4.7036 (PMC11033918; doi:10.1002/cam4.7036)
Supplement: Supplementary file 1 — Data S1. [file CAM4-13-e7036-s001.docx]

**Supplementary files**

**Search strategy in PubMed**

#6 Search: #4 AND #5 Sort by: Most Recent

1,249 09:39:12

#5 Search: random*[Title] Sort by: Most Recent

282,663 09:26:52

#4 Search: #1 OR #2 OR #3 Sort by: Most Recent

137,324 09:25:51

#3 Search: "Salivary Gland Neoplasm" OR "Cancer of Salivary Gland" OR "Salivary Gland Cancers" OR "Cancer of the Salivary Gland" OR "Salivary Gland Cancer" OR "Parotid Neoplasm" OR "Cancer of Parotid" OR "Parotid Cancers" OR "Parotid Cancer" OR "Cancer of the Parotid" OR "Sublingual Gland Neoplasms" OR "Submandibular Gland Neoplasm" OR "Submandibular Gland Neoplasms" Sort by: Most Recent

2,783 09:24:33

#2 Search: "Head and Neck Neoplasm" OR "Cancer of Head and Neck" OR "Head and Neck Cancer" OR "Cancer of the Head and Neck" OR "Upper Aerodigestive Tract Neoplasms" OR "UADT Neoplasms" OR "Upper Aerodigestive Tract Neoplasm" OR "Head Neoplasms" OR "Head Neoplasm" OR "neck Neoplasms" OR "Neck Neoplasm" OR "Cancer of Head" OR "Head Cancers" OR "Head Cancer" OR "Cancer of the Head" OR "Cancer of Neck" OR "Neck Cancers" OR "Neck Cancer" OR "Cancer of the Neck" Sort by: Most Recent

77,464 09:24:19

#1 Search: "Mouth Neoplasm" OR "mouth neoplasms" OR "oral neoplasm" OR "oral neoplasms" OR "Cancer of Mouth" OR "Mouth Cancer" OR "mouth cancers" OR "Oral Cancer" OR "oral cancers" OR "Cancer of the Mouth" OR "gingival neoplasm" OR "gingival neoplasms" OR "Lip Neoplasm" OR "Cancer of Lip" OR "Lip Cancers" OR "Cancer of the Lip" OR "Lip Cancer" OR "palatal neoplasm" OR "palatal neoplasms" OR "Jaw Neoplasm" OR "Cancer of the Jaw" OR "Jaw Cancer" OR "Maxillary Neoplasm" OR "Maxillary Neoplasms" OR "Mandibular Neoplasm" OR "Mandibular Neoplasms" Sort by: Most Recent

63,513 09:23:58

**List of included articles: (N = 204)**

***Articles 2000–2002 : (N = 20)***

[1]  Caponigro, F. et al. (2002). Cisplatin, raltitrexed, levofolinic acid and 5-fluorouracil in locally advanced or metastatic squamous cell carcinoma of the head and neck: a phase II randomized study. *Oncology*. DOI: 10.1159/000065470.

[2]  Scott, S.N. et al. (2002). The effects of epoetin alfa on transfusion requirements in head and neck cancer patients: a prospective, randomized, placebo-controlled study. *The Laryngoscope*. DOI: 10.1097/00005537-200207000-00015.

[3]  Evensen, J.F. et al. (2001). Effects of Na-sucrose octasulfate on skin and mucosa reactions during radiotherapy of head and neck cancers--a randomized prospective study. *Acta Oncologica (Stockholm, Sweden)*. DOI: 10.1080/02841860152619188.

[4]  Ehrnrooth, E. et al. (2001). Randomized trial of opioids versus tricyclic antidepressants for radiation-induced mucositis pain in head and neck cancer. *Acta Oncologica (Stockholm, Sweden)*. DOI: 10.1080/02841860152619179.

[5]  Corvò, R. et al. (2001). Alternating chemoradiotherapy versus partly accelerated radiotherapy in locally advanced squamous cell carcinoma of the head and neck: results from a phase III randomized trial. *Cancer*. DOI: 10.1002/1097-0142(20011201)92:11<2856::aid-cncr10132>3.0.co;2-6.

[6]  Lam, P. et al. (2001). Prospective randomized study of post-operative chemotherapy with levamisole and UFT for head and neck carcinoma. *European Journal of Surgical Oncology: The Journal of the European Society of Surgical Oncology and the British Association of Surgical Oncology*. DOI: 10.1053/ejso.2001.1171.

[7]  Gupta, N.K. and Swindell, R. (2001). Concomitant methotrexate and radiotherapy in advanced head and neck cancer: 15-year follow-up of a randomized clinical trial. *Clinical Oncology (Royal College of Radiologists (Great Britain))*. DOI: 10.1053/clon.2001.9286.

[8]  Ang, K.K. et al. (2001). Randomized trial addressing risk features and time factors of surgery plus radiotherapy in advanced head-and-neck cancer. *International Journal of Radiation Oncology, Biology, Physics*. DOI: 10.1016/s0360-3016(01)01690-x.

[9]  Epstein, J.B. et al. (2001). Benzydamine HCl for prophylaxis of radiation-induced oral mucositis: results from a multicenter, randomized, double-blind, placebo-controlled clinical trial. *Cancer*. DOI: 10.1002/1097-0142(20010815)92:4<875::aid-cncr1396>3.0.co;2-1.

[10]  Staar, S. et al. (2001). Intensified hyperfractionated accelerated radiotherapy limits the additional benefit of simultaneous chemotherapy--results of a multicentric randomized German trial in advanced head-and-neck cancer. *International Journal of Radiation Oncology, Biology, Physics*. DOI: 10.1016/s0360-3016(01)01544-9.

[11]  Poulsen, M.G. et al. (2001). A randomised trial of accelerated and conventional radiotherapy for stage III and IV squamous carcinoma of the head and neck: a Trans-Tasman Radiation Oncology Group Study. *Radiotherapy and Oncology: Journal of the European Society for Therapeutic Radiology and Oncology*. DOI: 10.1016/s0167-8140(01)00347-4.

[12]  Wijers, O.B. et al. (2001). Mucositis reduction by selective elimination of oral flora in irradiated cancers of the head and neck: a placebo-controlled double-blind randomized study. *International Journal of Radiation Oncology, Biology, Physics*. DOI: 10.1016/s0360-3016(01)01444-4.

[13]  Sangthawan, D. et al. (2001). Randomized double blind, placebo-controlled study of pilocarpine administered during head and neck irradiation to reduce xerostomia. *Journal of the Medical Association of Thailand = Chotmaihet Thangphaet*.

[14]  Mayne, S.T. et al. (2001). Randomized trial of supplemental beta-carotene to prevent second head and neck cancer. *Cancer Research*.

[15]  Grötz, K.A. et al. (2001). Prophylaxis of radiogenic sialadenitis and mucositis by coumarin/troxerutine in patients with head and neck cancer--a prospective,randomized, placebo-controlled, double-blind study. *The British Journal of Oral & Maxillofacial Surgery*. DOI: 10.1054/bjom.2000.0459.

[16]  Steuer-Vogt, M.K. et al. (2001). The effect of an adjuvant mistletoe treatment programme in resected head and neck cancer patients: a randomised controlled clinical trial. *European Journal of Cancer (Oxford, England: 1990)*. DOI: 10.1016/s0959-8049(00)00360-9.

[17]  Brizel, D.M. et al. (2000). Phase III randomized trial of amifostine as a radioprotector in head and neck cancer. *Journal of Clinical Oncology: Official Journal of the American Society of Clinical Oncology*. DOI: 10.1200/JCO.2000.18.19.3339.

[18]  Haddad, P. and Karimi, M. (2002). A randomized, double-blind, placebo-controlled trial of concomitant pilocarpine with head and neck irradiation for prevention of radiation-induced xerostomia. *Radiotherapy and Oncology: Journal of the European Society for Therapeutic Radiology and Oncology*. DOI: 10.1016/s0167-8140(02)00104-4.

[19]  Ramírez-Amador, V. et al. (2002). Intralesional vinblastine vs. 3% sodium tetradecyl sulfate for the treatment of oral Kaposi’s sarcoma. A double blind, randomized clinical trial. *Oral Oncology*. DOI: 10.1016/s1368-8375(01)00100-2.

[20]  Bartelink, H. et al. (2002). Concomitant cisplatin and radiotherapy in a conventional and modified fractionation schedule in locally advanced head and neck cancer: a randomised phase II EORTC trial. *European Journal of Cancer (Oxford, England: 1990)*. DOI: 10.1016/s0959-8049(01)00425-7.

***Articles 2010–2012: (N = 45)***

[1]  Tsukuda, M. et al. (2010). Randomized controlled phase II comparison study of concurrent chemoradiotherapy with docetaxel, cisplatin, and 5-fluorouracil versus CCRT with cisplatin, 5-fluorouracil, methotrexate and leucovorin in patients with locally advanced squamous cell carcinoma of the head and neck. *Cancer Chemotherapy and Pharmacology*. DOI: 10.1007/s00280-009-1217-0.

[2]  Rasch, C.R.N. et al. (2010). Intra-arterial versus intravenous chemoradiation for advanced head and neck cancer: Results of a randomized phase 3 trial. *Cancer*. DOI: 10.1002/cncr.24916.

[3]  Overgaard, J. et al. (2010). Five versus six fractions of radiotherapy per week for squamous-cell carcinoma of the head and neck (IAEA-ACC study): a randomised, multicentre trial. *The Lancet. Oncology*. DOI: 10.1016/S1470-2045(10)70072-3.

[4]  Wong, R.K. et al. (2010). Phase II Randomized Trial of Acupuncture-Like Transcutaneous Electrical Nerve Stimulation to Prevent Radiation-Induced Xerostomia in Head and Neck Cancer Patients. *Journal of the Society for Integrative Oncology*.

[5]  Saunders, M.I. et al. (2010). Mature results of a randomized trial of accelerated hyperfractionated versus conventional radiotherapy in head-and-neck cancer. *International Journal of Radiation Oncology, Biology, Physics*. DOI: 10.1016/j.ijrobp.2009.04.082.

[6]  Levin, V.A. et al. (2011). Randomized double-blind placebo-controlled trial of bevacizumab therapy for radiation necrosis of the central nervous system. *International Journal of Radiation Oncology, Biology, Physics*. DOI: 10.1016/j.ijrobp.2009.12.061.

[7]  Pfister, D.G. et al. (2010). Acupuncture for pain and dysfunction after neck dissection: results of a randomized controlled trial. *Journal of Clinical Oncology: Official Journal of the American Society of Clinical Oncology*. DOI: 10.1200/JCO.2009.26.9860.

[8]  Büntzel, J. et al. (2010). Limited effects of selenium substitution in the prevention of radiation-associated toxicities. results of a randomized study in head and neck cancer patients. *Anticancer Research*.

[9]  van der Molen, L. et al. (2011). A randomized preventive rehabilitation trial in advanced head and neck cancer patients treated with chemoradiotherapy: feasibility, compliance, and short-term effects. *Dysphagia*. DOI: 10.1007/s00455-010-9288-y.

[10]  Hoff, C.M. et al. (2011). The importance of haemoglobin level and effect of transfusion in HNSCC patients treated with radiotherapy--results from the randomized DAHANCA 5 study. *Radiotherapy and Oncology: Journal of the European Society for Therapeutic Radiology and Oncology*. DOI: 10.1016/j.radonc.2010.09.024.

[11]  Bardet, E. et al. (2011). Subcutaneous compared with intravenous administration of amifostine in patients with head and neck cancer receiving radiotherapy: final results of the GORTEC2000-02 phase III randomized trial. *Journal of Clinical Oncology: Official Journal of the American Society of Clinical Oncology*. DOI: 10.1200/JCO.2009.25.5638.

[12]  Gouvêa de Lima, A. et al. (2012). Oral mucositis prevention by low-level laser therapy in head-and-neck cancer patients undergoing concurrent chemoradiotherapy: a phase III randomized study. *International Journal of Radiation Oncology, Biology, Physics*. DOI: 10.1016/j.ijrobp.2010.10.012.

[13]  Lorch, J.H. et al. (2011). Induction chemotherapy with cisplatin and fluorouracil alone or in combination with docetaxel in locally advanced squamous-cell cancer of the head and neck: long-term results of the TAX 324 randomised phase 3 trial. *The Lancet. Oncology*. DOI: 10.1016/S1470-2045(10)70279-5.

[14]  Nutting, C.M. et al. (2011). Parotid-sparing intensity modulated versus conventional radiotherapy in head and neck cancer (PARSPORT): a phase 3 multicentre randomised controlled trial. *The Lancet. Oncology*. DOI: 10.1016/S1470-2045(10)70290-4.

[15]  De Luis, D.A. et al. (2010). A randomized double-blind clinical trial with two different doses of arginine enhanced enteral nutrition in postsurgical cancer patients. *European Review for Medical and Pharmacological Sciences*.

[16]  Ghadjar, P. et al. (2012). Concomitant cisplatin and hyperfractionated radiotherapy in locally advanced head and neck cancer: 10-year follow-up of a randomized phase III trial (SAKK 10/94). *International Journal of Radiation Oncology, Biology, Physics*. DOI: 10.1016/j.ijrobp.2010.11.067.

[17]  Salama, J.K. et al. (2011). A randomized phase II study of 5-fluorouracil, hydroxyurea, and twice-daily radiotherapy compared with bevacizumab plus 5-fluorouracil, hydroxyurea, and twice-daily radiotherapy for intermediate-stage and T4N0-1 head and neck cancers. *Annals of Oncology: Official Journal of the European Society for Medical Oncology*. DOI: 10.1093/annonc/mdq736.

[18]  Satheeshkumar, P S et al. (2010). Effectiveness of triclosan in the management of radiation-induced oral mucositis: a randomized clinical trial. *Journal of cancer research and therapeutics*. DOI:10.4103/0973-1482.77109

[19]  Huilgol, N.G. et al. (2010). Hyperthermia with radiation in the treatment of locally advanced head and neck cancer: a report of randomized trial. *Journal of Cancer Research and Therapeutics*. DOI: 10.4103/0973-1482.77101.

[20]  Silander, E. et al. (2012). Impact of prophylactic percutaneous endoscopic gastrostomy on malnutrition and quality of life in patients with head and neck cancer: a randomized study. *Head & Neck*. DOI: 10.1002/hed.21700.

[21]  Machiels, J.-P. et al. (2011). Zalutumumab plus best supportive care versus best supportive care alone in patients with recurrent or metastatic squamous-cell carcinoma of the head and neck after failure of platinum-based chemotherapy: an open-label, randomised phase 3 trial. *The Lancet. Oncology*. DOI: 10.1016/S1470-2045(11)70034-1.

[22]  Bonaparte, J.P. et al. (2011). Healing time of radial forearm free flap donor sites after preoperative tissue expansion: randomized controlled trial. *Journal of Otolaryngology - Head & Neck Surgery = Le Journal D’oto-Rhino-Laryngologie Et De Chirurgie Cervico-Faciale*.

[23]  Walen, S.G. et al. (2011). The utility of the harmonic scalpel in selective neck dissection: a prospective, randomized trial. *Otolaryngology--Head and Neck Surgery: Official Journal of American Academy of Otolaryngology-Head and Neck Surgery*. DOI: 10.1177/0194599811403874.

[24]  Sniezek, P.J. et al. (2011). A randomized controlled trial comparing acetaminophen, acetaminophen and ibuprofen, and acetaminophen and codeine for postoperative pain relief after Mohs surgery and cutaneous reconstruction. *Dermatologic Surgery: Official Publication for American Society for Dermatologic Surgery [et Al.]*. DOI: 10.1111/j.1524-4725.2011.02022.x.

[25]  Bardy, J. et al. (2012). A double-blind, placebo-controlled, randomised trial of active manuka honey and standard oral care for radiation-induced oral mucositis. *The British Journal of Oral & Maxillofacial Surgery*. DOI: 10.1016/j.bjoms.2011.03.005.

[26]  Le, Q.-T. et al. (2011). Palifermin reduces severe mucositis in definitive chemoradiotherapy of locally advanced head and neck cancer: a randomized, placebo-controlled study. *Journal of Clinical Oncology: Official Journal of the American Society of Clinical Oncology*. DOI: 10.1200/JCO.2010.32.4095.

[27]  Sharma, A. et al. (2012). Lactobacillus brevis CD2 lozenges reduce radiation- and chemotherapy-induced mucositis in patients with head and neck cancer: a randomized double-blind placebo-controlled study. *European Journal of Cancer (Oxford, England: 1990)*. DOI: 10.1016/j.ejca.2011.06.010.

[28]  Tortochaux, J. et al. (2011). Randomized phase III trial (GORTEC 98-03) comparing re-irradiation plus chemotherapy versus methotrexate in patients with recurrent or a second primary head and neck squamous cell carcinoma, treated with a palliative intent. *Radiotherapy and Oncology: Journal of the European Society for Therapeutic Radiology and Oncology*. DOI: 10.1016/j.radonc.2011.06.025.

[29]  Ackerstaff, A.H. et al. (2012). Five-year quality of life results of the randomized clinical phase III (RADPLAT) trial, comparing concomitant intra-arterial versus intravenous chemoradiotherapy in locally advanced head and neck cancer. *Head & Neck*. DOI: 10.1002/hed.21851.

[30]  Gregoire, V. et al. (2011). Gefitinib plus cisplatin and radiotherapy in previously untreated head and neck squamous cell carcinoma: a phase II, randomized, double-blind, placebo-controlled study. *Radiotherapy and Oncology: Journal of the European Society for Therapeutic Radiology and Oncology*. DOI: 10.1016/j.radonc.2011.07.008.

[31]  Del Campo, J.M. et al. (2011). Effects of lapatinib monotherapy: results of a randomised phase II study in therapy-naive patients with locally advanced squamous cell carcinoma of the head and neck. *British Journal of Cancer*. DOI: 10.1038/bjc.2011.237.

[32]  Bourhis, J. et al. (2011). Accelerated radiotherapy and concomitant high dose chemotherapy in non resectable stage IV locally advanced HNSCC: results of a GORTEC randomized trial. *Radiotherapy and Oncology: Journal of the European Society for Therapeutic Radiology and Oncology*. DOI: 10.1016/j.radonc.2011.07.006.

[33]  Robson, V. et al. (2012). Randomised controlled feasibility trial on the use of medical grade honey following microvascular free tissue transfer to reduce the incidence of wound infection. *The British Journal of Oral & Maxillofacial Surgery*. DOI: 10.1016/j.bjoms.2011.07.014.

[34]  Carvalho, P. a. G. et al. (2011). Evaluation of low-level laser therapy in the prevention and treatment of radiation-induced mucositis: a double-blind randomized study in head and neck cancer patients. *Oral Oncology*. DOI: 10.1016/j.oraloncology.2011.08.021.

[35]  Parikh, S. et al. (2012). A double blind randomised trial of IIb or not IIb neck dissections on electromyography, clinical examination, and questionnaire-based outcomes: a feasibility study. *The British Journal of Oral & Maxillofacial Surgery*. DOI: 10.1016/j.bjoms.2011.09.007.

[36]  Carnaby-Mann, G. et al. (2012). “Pharyngocise”: randomized controlled trial of preventative exercises to maintain muscle structure and swallowing function during head-and-neck chemoradiotherapy. *International Journal of Radiation Oncology, Biology, Physics*. DOI: 10.1016/j.ijrobp.2011.06.1954.

[37]  Li, M. et al. (2012). Phase II multicenter, randomized, double-blind study of recombinant mutated human tumor necrosis factor-α in combination with chemotherapies in cancer patients. *Cancer Science*. DOI: 10.1111/j.1349-7006.2011.02153.x.

[38]  Lund-Nielsen, B. et al. (2011). The effect of honey-coated bandages compared with silver-coated bandages on treatment of malignant wounds-a randomized study. *Wound Repair and Regeneration: Official Publication of the Wound Healing Society [and] the European Tissue Repair Society*. DOI: 10.1111/j.1524-475X.2011.00735.x.

[49]  Bourhis, J. et al. (2012). Concomitant chemoradiotherapy versus acceleration of radiotherapy with or without concomitant chemotherapy in locally advanced head and neck carcinoma (GORTEC 99-02): an open-label phase 3 randomised trial. *The Lancet. Oncology*. DOI: 10.1016/S1470-2045(11)70346-1.

[40]  Meng, Z. et al. (2012). Sham-controlled, randomised, feasibility trial of acupuncture for prevention of radiation-induced xerostomia among patients with nasopharyngeal carcinoma. *European Journal of Cancer (Oxford, England: 1990)*. DOI: 10.1016/j.ejca.2011.12.030.

[41]  Mortensen, H.R. et al. (2012). Prevalence and peak incidence of acute and late normal tissue morbidity in the DAHANCA 6&7 randomised trial with accelerated radiotherapy for head and neck cancer. *Radiotherapy and Oncology: Journal of the European Society for Therapeutic Radiology and Oncology*. DOI: 10.1016/j.radonc.2012.01.002.

[42]  Gautam, A.P. et al. (2012). Low level helium neon laser therapy for chemoradiotherapy induced oral mucositis in oral cancer patients - a randomized controlled trial. *Oral Oncology*. DOI: 10.1016/j.oraloncology.2012.03.008.

[43]  Kotz, T. et al. (2012). Prophylactic swallowing exercises in patients with head and neck cancer undergoing chemoradiation: a randomized trial. *Archives of Otolaryngology--Head & Neck Surgery*. DOI: 10.1001/archoto.2012.187.

[44]  Scott, S.E. et al. (2012). A randomised controlled trial of a pilot intervention to encourage early presentation of oral cancer in high risk groups. *Patient Education and Counseling*. DOI: 10.1016/j.pec.2012.03.015.

[45]  Krebber, A.-M.H. et al. (2012). Stepped care targeting psychological distress in head and neck and lung cancer patients: a randomized clinical trial. *BMC cancer*. DOI: 10.1186/1471-2407-12-173.

***Articles 2020–2022: (N = 139)***

[1]  Chang, Y.-T. et al. (2022). Effect of Total Intravenous Anesthesia on Postoperative Pulmonary Complications in Patients Undergoing Microvascular Reconstruction for Head and Neck Cancer: A Randomized Clinical Trial. *JAMA otolaryngology-- head & neck surgery*. DOI: 10.1001/jamaoto.2022.2552.

[2]  Mierzwa, M.L. et al. (2022). Randomized Phase II study of Physiologic MRI-directed Adaptive Radiation Boost in Poor Prognosis Head and Neck Cancer. *Clinical Cancer Research: An Official Journal of the American Association for Cancer Research*. DOI: 10.1158/1078-0432.CCR-22-1522.

[3]  Kashyap, K. et al. (2022). Scrambler Therapy Enhances Quality of Life in Cancer Patients in a Palliative Care Setting: A Randomised Controlled Trial. *Indian Journal of Palliative Care*. DOI: 10.25259/IJPC_94_2021.

[4]  Löser, A. et al. (2022). Impact of dosimetric factors on long-term percutaneous enteral gastrostomy (PEG) tube dependence in head and neck cancer patients after (chemo)radiotherapy-results from a prospective randomized trial. *Strahlentherapie Und Onkologie: Organ Der Deutschen Rontgengesellschaft ... [et Al]*. DOI: 10.1007/s00066-022-01992-5.

[5]  Nett, H. et al. (2022). A prospective randomized comparative trial evaluating postoperative nutritional intervention in patients with oral cancer. *Scientific Reports*. DOI: 10.1038/s41598-022-18292-8.

[6]  Starmer, H.M. et al. (2022). Head and Neck Virtual Coach: A Randomized Control Trial of Mobile Health as an Adjunct to Swallowing Therapy During Head and Neck Radiation. *Dysphagia*. DOI: 10.1007/s00455-022-10506-5.

[7]  Wallon, G. et al. (2022). Effect of intravenous lidocaine on pain after head and neck cancer surgery (ELICO trial): A randomised controlled trial. *European Journal of Anaesthesiology*. DOI: 10.1097/EJA.0000000000001712.

[8]  Chen, Y.-H. et al. (2022). A randomized controlled trial of scapular exercises with electromyography biofeedback in oral cancer patients with accessory nerve dysfunction. *Supportive Care in Cancer: Official Journal of the Multinational Association of Supportive Care in Cancer*. DOI: 10.1007/s00520-022-07263-4.

[9]  Paillaud, E. et al. (2022). Effectiveness of Geriatric Assessment-Driven Interventions on Survival and Functional and Nutritional Status in Older Patients with Head and Neck Cancer: A Randomized Controlled Trial (EGeSOR). *Cancers*. DOI: 10.3390/cancers14133290.

[10]  Held, T. et al. (2022). Ways to unravel the clinical potential of carbon ions for head and neck cancer reirradiation: dosimetric comparison and local failure pattern analysis as part of the prospective randomized CARE trial. *Radiation Oncology*. DOI: 10.1186/s13014-022-02093-4.

[11]  Tungkasamit, T. et al. (2022). Reduction in severity of radiation-induced dermatitis in head and neck cancer patients treated with topical aloe vera gel: A randomized multicenter double-blind placebo-controlled trial. *European Journal of Oncology Nursing: The Official Journal of European Oncology Nursing Society*. DOI: 10.1016/j.ejon.2022.102164.

[12]  Sterba, K.R. et al. (2022). A pilot randomized controlled trial to evaluate a survivorship care planning intervention for head and neck cancer survivor-caregiver dyads. *Journal of Cancer Survivorship: Research and Practice*. DOI: 10.1007/s11764-022-01227-7.

[13]  Anderson, C.M. et al. (2022). Two-Year Tumor Outcomes of Phase 2B, Randomized, Double-Blind Trial of Avasopasem Manganese (GC4419) Versus Placebo to Reduce Severe Oral Mucositis Owing to Concurrent Radiation Therapy and Cisplatin for Head and Neck Cancer. *International Journal of Radiation Oncology, Biology, Physics*. DOI: 10.1016/j.ijrobp.2022.06.063.

[14]  Thambamroong, T. et al. (2022). Efficacy of Curcumin on Treating Cancer Anorexia-Cachexia Syndrome in Locally or Advanced Head and Neck Cancer: A Double-Blind, Placebo-Controlled Randomised Phase IIa Trial (CurChexia). *Journal of Nutrition and Metabolism*. DOI: 10.1155/2022/5425619.

[15]  Jiang, N. et al. (2022). Effects of an integrated supportive program on xerostomia and saliva characteristics in patients with head and neck cancer radiated with a low dose to the major salivary glands: a randomized controlled trial. *BMC oral health*. DOI: 10.1186/s12903-022-02225-y.

[16]  Min, S.-H. et al. (2022). Effects of ischemic conditioning on head and neck free flap oxygenation: a randomized controlled trial | Scientific Reports. *Scientific Reports*. DOI: 10.1038/s41598-022-12374-3.

[17]  Kuhn-Dall’Magro, A. et al. (2022). Low-level Laser Therapy in the Management of Oral Mucositis Induced by Radiotherapy: A Randomized Double-blind Clinical Trial. *The Journal of Contemporary Dental Practice*.

[18]  Chou, Y.-H. et al. (2022). Acupoint stimulation improves pain and quality of life in head and neck cancer patients with chemoradiotherapy: A randomized controlled trial. *Asia-Pacific Journal of Oncology Nursing*. DOI: 10.1016/j.apjon.2021.11.002.

[19]  Lynggaard, C.D. et al. (2022). Long-term Safety of Treatment with Autologous Mesenchymal Stem Cells in Patients with Radiation-Induced Xerostomia: Primary Results of the MESRIX Phase I/II Randomized Trial. *Clinical Cancer Research: An Official Journal of the American Association for Cancer Research*. DOI: 10.1158/1078-0432.CCR-21-4520.

[20]  Palma, D.A. et al. (2022). Assessment of Toxic Effects and Survival in Treatment Deescalation With Radiotherapy vs Transoral Surgery for HPV-Associated Oropharyngeal Squamous Cell Carcinoma: The ORATOR2 Phase 2 Randomized Clinical Trial. *JAMA oncology*. DOI: 10.1001/jamaoncol.2022.0615.

[21]  Takahashi, M. et al. (2022). Quality of Life Analysis of HPV-Positive Oropharyngeal Cancer Patients in a Randomized Trial of Reduced-Dose Versus Standard Chemoradiotherapy: 5-Year Follow-Up. *Frontiers in Oncology*. DOI: 10.3389/fonc.2022.859992.

[22]  Mohamed Elfadil, O. et al. (2022). Safety and effectiveness of radiologic and endoscopic percutaneous gastrostomy placement: A randomized study. *JPEN. Journal of parenteral and enteral nutrition*. DOI: 10.1002/jpen.2365.

[23]  Persson, K. et al. (2022). Pain management with popliteal block for fibular graft harvesting in head and neck reconstruction; a randomised double-blind placebo-controlled study. *Oral Oncology*. DOI: 10.1016/j.oraloncology.2022.105833.

[24]  Welz, S. et al. (2022). Dose escalation to hypoxic subvolumes in head and neck cancer: A randomized phase II study using dynamic [18F]FMISO PET/CT. *Radiotherapy and Oncology: Journal of the European Society for Therapeutic Radiology and Oncology*. DOI: 10.1016/j.radonc.2022.03.021.

[25]  Patil, V. et al. (2022). RMAC study: A randomized study for evaluation of metronomic adjuvant chemotherapy in recurrent head and neck cancers post salvage surgical resection in those who are ineligible for re-irradiation. *Oral Oncology*. DOI: 10.1016/j.oraloncology.2022.105816.

[26]  Atar, S. et al. (2022). The efficacy of Kinesio taping on lymphedema following head and neck cancer therapy: a randomized, double blind, sham-controlled trial. *Physiotherapy Theory and Practice*. DOI: 10.1080/09593985.2022.2056862.

[27]  Linxweiler, M. et al. (2022). Cytology-based Cancer Surgery of the Head and Neck (CyCaS-HN): a prospective, randomized, controlled clinical trial. *European archives of oto-rhino-laryngology: official journal of the European Federation of Oto-Rhino-Laryngological Societies (EUFOS): affiliated with the German Society for Oto-Rhino-Laryngology - Head and Neck Surgery*. DOI: 10.1007/s00405-022-07333-7.

[28]  Wu, H.-Y. et al. (2022). Preoperative oral carbohydrates in elderly patients undergoing free flap surgery for oral cancer: randomized controlled trial. *International Journal of Oral and Maxillofacial Surgery*. DOI: 10.1016/j.ijom.2022.02.014.

[29]  Brahmanandan, R. et al. (2022). A randomized control trial to study the effect of bilateral Gow-gates block on visibility of surgical field and hemodynamic response in patients with carcinoma tongue. *Cancer Treatment and Research Communications*. DOI: 10.1016/j.ctarc.2022.100535.

[30]  Kiyota, N. et al. (2022). Weekly Cisplatin Plus Radiation for Postoperative Head and Neck Cancer (JCOG1008): A Multicenter, Noninferiority, Phase II/III Randomized Controlled Trial. *Journal of Clinical Oncology*. DOI: 10.1200/JCO.21.01293.

[31]  Kongwattanakul, S. et al. (2022). Prophylactic management of radiation-induced mucositis using herbal mouthwash in patients with head and neck cancer: an assessor-blinded randomized controlled trial. *Journal of Complementary & Integrative Medicine*. DOI: 10.1515/jcim-2021-0457.

[32]  Pakkanen, P. et al. (2022). Survival and Larynx Preservation in Early Glottic Cancer: A Randomized Trial Comparing Laser Surgery and Radiation Therapy. *International Journal of Radiation Oncology, Biology, Physics*. DOI: 10.1016/j.ijrobp.2022.01.010.

[33]  Maleki, S. et al. (2022). A randomised controlled trial of clinical pharmacy intervention versus standard care to improve medication adherence in outpatients with head and neck cancer receiving radiotherapy. *Supportive Care in Cancer: Official Journal of the Multinational Association of Supportive Care in Cancer*. DOI: 10.1007/s00520-021-06779-5.

[34]  Mortensen, A. et al. (2022). Needs assessment in patients surgically treated for head and neck cancer-a randomized controlled trial. *Supportive Care in Cancer: Official Journal of the Multinational Association of Supportive Care in Cancer*. DOI: 10.1007/s00520-021-06759-9.

[35]  Tuomi, L. et al. (2022). The effect of the Shaker head-lift exercise on swallowing function following treatment for head and neck cancer: Results from a randomized, controlled trial with videofluoroscopic evaluation. *Head & Neck*. DOI: 10.1002/hed.26982.

[36]  Dodd, M.J. et al. (2022). A randomized clinical trial of granulocyte macrophage colony stimulating factor mouthwash for oral mucositis in head and neck cancer. *European Journal of Oncology Nursing: The Official Journal of European Oncology Nursing Society*. DOI: 10.1016/j.ejon.2022.102093.

[37]  Dechaphunkul, T. et al. (2022). Benefits of immunonutrition in patients with head and neck cancer receiving chemoradiation: A phase II randomized, double-blind study. *Clinical Nutrition*. DOI: 10.1016/j.clnu.2021.12.035.

[38]  Babu, M.J. et al. (2021). Effect of Two Different Tranexamic Acid Doses on Blood Loss in Head and Neck Cancer Surgery: A Randomized, Double-Blind, Controlled Study. *Cureus*. DOI: 10.7759/cureus.20190.

[39]  Ashour, M.G. et al. (2022). Swallowing sparing intensity modulated radiotherapy versus standard parotid sparing intensity-modulated radiotherapy for treatment of head and neck cancer: a randomized clinical trial. *Acta Oncologica (Stockholm, Sweden)*. DOI: 10.1080/0284186X.2021.2022198.

[40]  Nichols, A.C. et al. (2022). Randomized Trial of Radiotherapy Versus Transoral Robotic Surgery for Oropharyngeal Squamous Cell Carcinoma: Long-Term Results of the ORATOR Trial. *Journal of Clinical Oncology: Official Journal of the American Society of Clinical Oncology*. DOI: 10.1200/JCO.21.01961.

[41]  Turkdogan, S. et al. (2022). Effect of Perioperative Patient Education via Animated Videos in Patients Undergoing Head and Neck Surgery: A Randomized Clinical Trial. *JAMA otolaryngology-- head & neck surgery*. DOI: 10.1001/jamaoto.2021.3765.

[42]  Suppadungsuk, S. et al. (2022). Preloading magnesium attenuates cisplatin-associated nephrotoxicity: pilot randomized controlled trial (PRAGMATIC study). *ESMO open*. DOI: 10.1016/j.esmoop.2021.100351.

[43]  Lin, Y. et al. (2022). Apatinib vs Placebo in Patients With Locally Advanced or Metastatic, Radioactive Iodine-Refractory Differentiated Thyroid Cancer: The REALITY Randomized Clinical Trial. *JAMA oncology*. DOI: 10.1001/jamaoncol.2021.6268.

[44]  Chaukar, D. et al. (2022). Prospective Phase II Open-Label Randomized Controlled Trial to Compare Mandibular Preservation in Upfront Surgery With Neoadjuvant Chemotherapy Followed by Surgery in Operable Oral Cavity Cancer. *Journal of Clinical Oncology: Official Journal of the American Society of Clinical Oncology*. DOI: 10.1200/JCO.21.00179.

[45]  Kutz, L.M. et al. (2022). Quality of life, HPV-status and phase angle predict survival in head and neck cancer patients under (chemo)radiotherapy undergoing nutritional intervention: Results from the prospective randomized HEADNUT-trial. *Radiotherapy and Oncology: Journal of the European Society for Therapeutic Radiology and Oncology*. DOI: 10.1016/j.radonc.2021.11.011.

[46]  Cook, A. et al. (2022). Randomized Phase 3, Double-Blind, Placebo-Controlled Study of Prophylactic Gabapentin for the Reduction of Oral Mucositis Pain During the Treatment of Oropharyngeal Squamous Cell Carcinoma. *International Journal of Radiation Oncology, Biology, Physics*. DOI: 10.1016/j.ijrobp.2021.11.012.

[47]  Wennerberg, J. et al. (2022). Results from a prospective, randomised study on (accelerated) preoperative versus (conventional) postoperative radiotherapy in treatment of patients with resectable squamous cell carcinoma of the oral cavity - The ARTSCAN 2 study. *Radiotherapy and Oncology: Journal of the European Society for Therapeutic Radiology and Oncology*. DOI: 10.1016/j.radonc.2021.11.008.

[48]  Loh, E.-W. et al. (2022). Effect of progressive muscle relaxation on postoperative pain, fatigue, and vital signs in patients with head and neck cancers: A randomized controlled trial. *Patient Education and Counseling*. DOI: 10.1016/j.pec.2021.10.034.

[49]  Zhang, X. et al. (2021). Radiotherapy for head and neck tumours using an oral fixation and parameter acquisition device and TOMO technology: a randomised controlled study. *BMJ open*. DOI: 10.1136/bmjopen-2021-052542.

[50]  Castro-Martín, E. et al. (2021). Myofascial Induction Therapy Improves the Sequelae of Medical Treatment in Head and Neck Cancer Survivors: A Single-Blind, Placebo-Controlled, Randomized Cross-Over Study. *Journal of Clinical Medicine*. DOI: 10.3390/jcm10215003.

[51]  Kauark-Fontes, E. et al. (2022). Extraoral photobiomodulation for prevention of oral and oropharyngeal mucositis in head and neck cancer patients: interim analysis of a randomized, double-blind, clinical trial. *Supportive Care in Cancer: Official Journal of the Multinational Association of Supportive Care in Cancer*. DOI: 10.1007/s00520-021-06625-8.

[52]  Arantes, D.A.C. et al. (2021). Safety and efficacy of a mucoadhesive phytomedication containing curcuminoids and Bidens pilosa L. extract in the prevention and treatment of radiochemotherapy-induced oral mucositis: Triple-blind, randomized, placebo-controlled, clinical trial. *Head & Neck*. DOI: 10.1002/hed.26892.

[53]  Steenbakkers, R.J.H.M. et al. (2022). Parotid Gland Stem Cell Sparing Radiation Therapy for Patients With Head and Neck Cancer: A Double-Blind Randomized Controlled Trial. *International Journal of Radiation Oncology, Biology, Physics*. DOI: 10.1016/j.ijrobp.2021.09.023.

[54]  Khantwal, G. et al. (2021). Effect of Postsurgical Nurse-led Follow-ups on Quality of Life in Head-and-Neck Cancer Patients: A Pilot Randomized Controlled Trial. *Asia-Pacific Journal of Oncology Nursing*. DOI: 10.4103/apjon.apjon-2112.

[55]  Kia, S.J. et al. (2021). Effects of nanomicelle curcumin capsules on prevention and treatment of oral mucosits in patients under chemotherapy with or without head and neck radiotherapy: a randomized clinical trial. *BMC complementary medicine and therapies*. DOI: 10.1186/s12906-021-03400-4.

[56]  Klinghammer, K. et al. (2021). A randomized phase II study comparing the efficacy and safety of the glyco-optimized anti-EGFR antibody tomuzotuximab against cetuximab in patients with recurrent and/or metastatic squamous cell cancer of the head and neck - the RESGEX study. *ESMO open*. DOI: 10.1016/j.esmoop.2021.100242.

[57]  Yang, Q. et al. (2021). The impact of induction chemotherapy on long-term quality of life in patients with locoregionally advanced nasopharyngeal carcinoma: Outcomes from a randomised phase 3 trial. *Oral Oncology*. DOI: 10.1016/j.oraloncology.2021.105494.

[58]  Guo, Y. et al. (2021). First-line treatment with chemotherapy plus cetuximab in Chinese patients with recurrent and/or metastatic squamous cell carcinoma of the head and neck: Efficacy and safety results of the randomised, phase III CHANGE-2 trial. *European Journal of Cancer (Oxford, England: 1990)*. DOI: 10.1016/j.ejca.2021.06.039.

[59]  Chen, Y.-H. et al. (2021). Effects of Conscious Control of Scapular Orientation in Oral Cancer Survivors With Scapular Dyskinesis: A Randomized Controlled Trial. *Integrative Cancer Therapies*. DOI: 10.1177/15347354211040827.

[60]  Rosenthal, M. et al. (2021). Flexible fiber-based CO2 laser vs monopolar cautery for resection of oral cavity lesions: A single center randomized controlled trial assessing pain and quality of life following surgery. *Laryngoscope Investigative Otolaryngology*. DOI: 10.1002/lio2.572.

[61]  Sittitrai, P. et al. (2021). Effect of a perioperative immune-enhancing diet in clean-contaminated head and neck cancer surgery: A randomized controlled trial. *International Journal of Surgery (London, England)*. DOI: 10.1016/j.ijsu.2021.106051.

[62]  Billa, O. et al. (2021). Randomized Trial Assessing the Impact of Routine Assessment of Health-Related Quality of Life in Patients with Head and Neck Cancer. *Cancers*. DOI: 10.3390/cancers13153826.

[63]  Ghonaim, E. et al. (2021). Possible protective effect of pantoprazole against cisplatin-induced nephrotoxicity in head and neck cancer patients: a randomized controlled trial. *Medical Oncology (Northwood, London, England)*. DOI: 10.1007/s12032-021-01558-y.

[64]  Iacovelli, N.A. et al. (2021). A Randomized, Double-Blind, Placebo-Controlled, Cross-Over Study to Evaluate the Efficacy of AqualiefTM Mucoadhesive Tablets in Head and Neck Cancer Patients Who Developed Radiation-Induced Xerostomia. *Cancers*. DOI: 10.3390/cancers13143456.

[65]  Nutting, C.M. et al. (2021). Dose-escalated intensity-modulated radiotherapy in patients with locally advanced laryngeal and hypopharyngeal cancers: ART DECO, a phase III randomised controlled trial. *European Journal of Cancer (Oxford, England: 1990)*. DOI: 10.1016/j.ejca.2021.05.021.

[66]  Ben-Arie, E. et al. (2021). Digestion-Specific Acupuncture Effect on Feeding Intolerance in Critically Ill Post-Operative Oral and Hypopharyngeal Cancer Patients: A Single-Blind Randomized Control Trial. *Nutrients*. DOI: 10.3390/nu13062110.

[67]  Liao, Y.-C. et al. (2021). Effectiveness of green tea mouthwash for improving oral health status in oral cancer patients: A single-blind randomized controlled trial. *International Journal of Nursing Studies*. DOI: 10.1016/j.ijnurstu.2021.103985.

[68]  Soni, T.P. et al. (2022). A Randomized, Placebo-Controlled Study to Evaluate the Effect of Bio-Enhanced Turmeric Formulation on Radiation-Induced Oral Mucositis. *ORL; journal for oto-rhino-laryngology and its related specialties*. DOI: 10.1159/000516577.

[69]  Ren, G. et al. (2021). A multicenter randomized phase II trial of hyperthermia combined with TPF induction chemotherapy compared with TPF induction chemotherapy in locally advanced resectable oral squamous cell carcinoma. *International Journal of Hyperthermia: The Official Journal of European Society for Hyperthermic Oncology, North American Hyperthermia Group*. DOI: 10.1080/02656736.2021.1937714.

[70]  Yokota, T. et al. (2021). Phase 3 Randomized Trial of Topical Steroid Versus Placebo for Prevention of Radiation Dermatitis in Patients With Head and Neck Cancer Receiving Chemoradiation. *International Journal of Radiation Oncology, Biology, Physics*. DOI: 10.1016/j.ijrobp.2021.05.133.

[71]  Ameri, A. et al. (2022). Randomized trial on acute toxicities of weekly vs three-weekly cisplatin-based chemoradiation in head and neck cancer. *Cancer Reports (Hoboken, N.J.)*. DOI: 10.1002/cnr2.1425.

[72]  Rischin, D. et al. (2021). Randomized Trial of Radiation Therapy With Weekly Cisplatin or Cetuximab in Low-Risk HPV-Associated Oropharyngeal Cancer (TROG 12.01) - A Trans-Tasman Radiation Oncology Group Study. *International Journal of Radiation Oncology, Biology, Physics*. DOI: 10.1016/j.ijrobp.2021.04.015.

[73]  Zheng, Y. et al. (2021). Efficacy and Safety of Cetuximab Plus Cisplatin Alone or in Combination With Paclitaxel in Patients With Head and Neck Squamous Cell Carcinoma: A Randomized Trial. *Cancer Control: Journal of the Moffitt Cancer Center*. DOI: 10.1177/1073274821997444.

[74]  Keil, F. et al. (2021). Docetaxel, cisplatin and 5-FU compared with docetaxel, cisplatin and cetuximab as induction chemotherapy in advanced squamous cell carcinoma of the head and neck: Results of a randomised phase II AGMT trial. *European Journal of Cancer (Oxford, England: 1990)*. DOI: 10.1016/j.ejca.2021.03.051.

[75]  Jansen, F. et al. (2021). Cost-utility and cost-effectiveness of a guided self-help head and neck exercise program for patients treated with total laryngectomy: Results of a multi-center randomized controlled trial. *Oral Oncology*. DOI: 10.1016/j.oraloncology.2021.105306.

[76]  Beck, A.K. et al. (2021). Is fidelity to a complex behaviour change intervention associated with patient outcomes? Exploring the relationship between dietitian adherence and competence and the nutritional status of intervention patients in a successful stepped-wedge randomised clinical trial of eating as treatment (EAT). *Implementation Science*. DOI: 10.1186/s13012-021-01118-y.

[77]  Hasegawa, Y. et al. (2021). Neck Dissections Based on Sentinel Lymph Node Navigation Versus Elective Neck Dissections in Early Oral Cancers: A Randomized, Multicenter, and Noninferiority Trial. *Journal of Clinical Oncology*. DOI: 10.1200/JCO.20.03637.

[78]  Held, T. et al. (2021). 3D-printed individualized tooth-borne tissue retraction devices compared to conventional dental splints for head and neck cancer radiotherapy: a randomized controlled trial. *Radiation Oncology*. DOI: 10.1186/s13014-021-01803-8.

[79]  Ambulkar, R. et al. (2020). A randomized controlled trial comparing McGRATH series 5 videolaryngoscope with the Macintosh laryngoscope for nasotracheal intubation. *Journal of Anaesthesiology, Clinical Pharmacology*. DOI: 10.4103/joacp.JOACP_1_20.

[80]  Sun, L. et al. (2022). Narrative therapy to relieve stigma in oral cancer patients: A randomized controlled trial. *International Journal of Nursing Practice*. DOI: 10.1111/ijn.12926.

[81]  Dymackova, R. et al. (2021). Effect of Acupuncture in Pain Management of Head and Neck Cancer Radiotherapy: Prospective Randomized Unicentric Study. *Journal of Clinical Medicine*. DOI: 10.3390/jcm10051111.

[82]  Grover, A. et al. (2021). A randomized prospective study comparing acute toxicity, compliance and objective response rate between simultaneous integrated boost and sequential intensity-modulated radiotherapy for locally advanced head and neck cancer. *Radiation Oncology Journal*. DOI: 10.3857/roj.2020.01018.

[83]  Agha-Hosseini, F. et al. (2021). Mouthwash Containing Vitamin E, Triamcinolon, and Hyaluronic Acid Compared to Triamcinolone Mouthwash Alone in Patients With Radiotherapy-Induced Oral Mucositis: Randomized Clinical Trial. *Frontiers in Oncology*. DOI: 10.3389/fonc.2021.614877.

[84]  Löser, A. et al. (2021). Head and neck cancer patients under (chemo-)radiotherapy undergoing nutritional intervention: Results from the prospective randomized HEADNUT-trial. *Radiotherapy and Oncology: Journal of the European Society for Therapeutic Radiology and Oncology*. DOI: 10.1016/j.radonc.2021.03.019.

[85]  Lozano, A. et al. (2021). Randomized placebo-controlled phase II trial of high-dose melatonin mucoadhesive oral gel for the prevention and treatment of oral mucositis in patients with head and neck cancer undergoing radiation therapy concurrent with systemic treatment. *Clinical & Translational Oncology: Official Publication of the Federation of Spanish Oncology Societies and of the National Cancer Institute of Mexico*. DOI: 10.1007/s12094-021-02586-w.

[86]  Adkins, D.R. et al. (2021). Palbociclib and cetuximab compared with placebo and cetuximab in platinum-resistant, cetuximab-naïve, human papillomavirus-unrelated recurrent or metastatic head and neck squamous cell carcinoma: A double-blind, randomized, phase 2 trial. *Oral Oncology*. DOI: 10.1016/j.oraloncology.2021.105192.

[87]  Lin, K.-Y. et al. (2021). Effects of Exercise in Patients Undergoing Chemotherapy for Head and Neck Cancer: A Pilot Randomized Controlled Trial. *International Journal of Environmental Research and Public Health*. DOI: 10.3390/ijerph18031291.

[88]  Nagaoka, H. et al. (2021). Effects of an Indomethacin Oral Spray on Pain Due to Oral Mucositis in Cancer Patients Treated With Radiotherapy and Chemotherapy: A Double-Blind, Randomized, Placebo-Controlled Trial (JORTC-PAL04). *Journal of Pain and Symptom Management*. DOI: 10.1016/j.jpainsymman.2021.01.123.

[89]  Ameri, A. et al. (2021). Effect of Honey-Lemon Spray Versus Benzydamine Hydrochloride Spray on Radiation-Induced Acute Oral Mucositis in Head and Neck Cancer Patients: A Pilot, Randomized, Double-Blind, Active-Controlled Clinical Trial. *Journal of Alternative and Complementary Medicine (New York, N.Y.)*. DOI: 10.1089/acm.2020.0468.

[90]  Schutte, L.E.R. et al. (2021). Effect of Stepped Care on Sexual Interest and Enjoyment in Distressed Patients with Head and Neck Cancer: A Randomized Controlled Trial. *Sexual Medicine*. DOI: 10.1016/j.esxm.2020.100304.

[91]  Eriksson, H. et al. (2021). Voice Outcomes Following Head-Lift Exercises in Head and Neck Cancer: A Randomized Controlled Study. *Journal of Voice: Official Journal of the Voice Foundation*. DOI: 10.1016/j.jvoice.2020.12.015.

[92]  Shah, S. et al. (2020). Effectiveness of curcumin mouthwash on radiation-induced oral mucositis among head and neck cancer patients: A triple-blind, pilot randomised controlled trial. *Indian Journal of Dental Research: Official Publication of Indian Society for Dental Research*. DOI: 10.4103/ijdr.IJDR_822_18.

[93]  Bouleftour, W. et al. (2021). Effectiveness of a nurse-led telephone follow-up in the therapeutic management of patients receiving oral antineoplastic agents: a randomized, multicenter controlled trial (ETICCO study). *Supportive Care in Cancer: Official Journal of the Multinational Association of Supportive Care in Cancer*. DOI: 10.1007/s00520-020-05955-3.

[94]  Aladashi, O.Q.S. et al. (2021). Effect of submental flap reconstruction versus obturator rehabilitation after maxillectomy on quality of life: a randomized clinical trial. *International Journal of Oral and Maxillofacial Surgery*. DOI: 10.1016/j.ijom.2020.12.008.

[95]  Soutome, S. et al. (2021). Effects of a bioadhesive barrier-forming oral liquid on pain due to radiation-induced oral mucositis in patients with head and neck cancer: A randomized crossover, preliminary study. *Journal of Dental Sciences*. DOI: 10.1016/j.jds.2020.07.006.

[96]  de Carvalho, C.S. et al. (2021). Preoperative Fasting Abbreviation With Whey Protein Reduces the Occurrence of Postoperative Complications in Patients With Head and Neck Cancer: A Randomized Clinical Trial. *Nutrition in Clinical Practice: Official Publication of the American Society for Parenteral and Enteral Nutrition*. DOI: 10.1002/ncp.10624.

[97]  Balbinot, J. et al. (2022). Quality of life in tongue cancer treated patients before and after speech therapy: a randomized clinical trial. *Brazilian Journal of Otorhinolaryngology*. DOI: 10.1016/j.bjorl.2020.10.005.

[98]  Smaily, H. et al. (2021). Smoking cessation intervention for patients with head and neck cancer: A prospective randomized controlled trial. *American Journal of Otolaryngology*. DOI: 10.1016/j.amjoto.2020.102832.

[99]  Tao, Y. et al. (2020). Avelumab-cetuximab-radiotherapy versus standards of care in locally advanced squamous-cell carcinoma of the head and neck: The safety phase of a randomised phase III trial GORTEC 2017-01 (REACH). *European Journal of Cancer (Oxford, England: 1990)*. DOI: 10.1016/j.ejca.2020.09.008.

[100]  Gremore, T.M. et al. (2021). Couple-based communication intervention for head and neck cancer: a randomized pilot trial. *Supportive Care in Cancer: Official Journal of the Multinational Association of Supportive Care in Cancer*. DOI: 10.1007/s00520-020-05848-5.

[101]  Kumar, P.S. et al. (2020). A Randomized Control Trial to Assess Intraoperative and Postoperative Outcomes of Colorado Microdissection Needle Versus Conventional Surgical Knife in Neck Dissection. *Journal of Maxillofacial and Oral Surgery*. DOI: 10.1007/s12663-020-01377-0.

[102]  Garrel, R. et al. (2020). Equivalence Randomized Trial to Compare Treatment on the Basis of Sentinel Node Biopsy Versus Neck Node Dissection in Operable T1-T2N0 Oral and Oropharyngeal Cancer. *Journal of Clinical Oncology*. DOI: 10.1200/JCO.20.01661.

[103]  Durham, J.S. et al. (2020). Effect of Fluorescence Visualization-Guided Surgery on Local Recurrence of Oral Squamous Cell Carcinoma: A Randomized Clinical Trial. *JAMA otolaryngology-- head & neck surgery*. DOI: 10.1001/jamaoto.2020.3147.

[104]  Kochhar, A. et al. (2020). Cervical epidural analgesia combined with general anesthesia for head and neck cancer surgery: A randomized study. *Journal of Anaesthesiology, Clinical Pharmacology*. DOI: 10.4103/joacp.JOACP_72_19.

[105]  Martins, A.F.L. et al. (2021). The Effect of Photobiomodulation on Nitrite and Inflammatory Activity in Radiotherapy-Induced Oral Mucositis: A Randomized Clinical Trial. *Lasers in Surgery and Medicine*. DOI: 10.1002/lsm.23328.

[106]  Bakr, I.S. et al. (2021). Vitamin D oral gel for prevention of radiation-induced oral mucositis: A randomized clinical trial. *Oral Diseases*. DOI: 10.1111/odi.13650.

[107]  Ebert, N. et al. (2021). Results of a randomized controlled phase III trial: efficacy of polyphenol-containing cystus® tea mouthwash solution for the reduction of mucositis in head and neck cancer patients undergoing external beam radiotherapy. *Strahlentherapie Und Onkologie: Organ Der Deutschen Rontgengesellschaft ... [et Al]*. DOI: 10.1007/s00066-020-01684-y.

[108]  Kashyap, K. et al. (2020). The Efficacy of Scrambler Therapy for the Management of Head, Neck and Thoracic Cancer Pain: A Randomized Controlled Trial. *Pain Physician*.

[109]  Wozniak, G. et al. (2020). Randomised clinical trial on 7-days-a-week postoperative radiotherapy vs. concurrent postoperative radio-chemotherapy in locally advanced cancer of the oral cavity/oropharynx. *The British Journal of Radiology*. DOI: 10.1259/bjr.20200288.

[110]  Gupta, T. et al. (2020). Intensity-modulated radiation therapy versus three-dimensional conformal radiotherapy in head and neck squamous cell carcinoma: long-term and mature outcomes of a prospective randomized trial. *Radiation Oncology (London, England)*. DOI: 10.1186/s13014-020-01666-5.

[111]  Deschuymer, S. et al. (2021). Randomized Clinical Trial on Reduction of Radiotherapy Dose to the Elective Neck in Head and Neck Squamous Cell Carcinoma: Results on the Quality of Life. *Quality of Life Research: An International Journal of Quality of Life Aspects of Treatment, Care and Rehabilitation*. DOI: 10.1007/s11136-020-02628-w.

[112]  Zhao, Z. et al. (2020). Single-Agent Versus Double-Agent Chemotherapy in Concurrent Chemoradiotherapy for Esophageal Squamous Cell Carcinoma: Prospective, Randomized, Multicenter Phase II Clinical Trial. *The Oncologist*. DOI: 10.1634/theoncologist.2020-0808.

[113]  Thomas, A. et al. (2020). Effect of Muscle Energy Techniques V/S Active Range of Motion Exercises on Shoulder Function Post Modified Radical Neck Dissection in patients with Head and Neck Cancer - A Randomized Clinical Trial. *Asian Pacific journal of cancer prevention: APJCP*. DOI: 10.31557/APJCP.2020.21.8.2389.

[114]  Menon, A. et al. (2021). Topical Betamethasone Valerate As a Prophylactic Agent to Prevent Acute Radiation Dermatitis in Head and Neck Malignancies: A Randomized, Open-Label, Phase 3 Trial. *International Journal of Radiation Oncology, Biology, Physics*. DOI: 10.1016/j.ijrobp.2020.08.040.

[115]  Schoenfeld, J.D. et al. (2020). Neoadjuvant Nivolumab or Nivolumab Plus Ipilimumab in Untreated Oral Cavity Squamous Cell Carcinoma: A Phase 2 Open-Label Randomized Clinical Trial. *JAMA oncology*. DOI: 10.1001/jamaoncol.2020.2955.

[116]  Tousif, D. et al. (2020). Randomized Controlled Study Comparing Efficacy and Toxicity of Weekly vs. 3-Weekly Induction Chemotherapy in Locally Advanced Head and Neck Squamous Cell Carcinoma. *Frontiers in Oncology*. DOI: 10.3389/fonc.2020.01284.

[117]  Patil, V. et al. (2020). Low-cost oral metronomic chemotherapy versus intravenous cisplatin in patients with recurrent, metastatic, inoperable head and neck carcinoma: an open-label, parallel-group, non-inferiority, randomised, phase 3 trial. *The Lancet. Global Health*. DOI: 10.1016/S2214-109X(20)30275-8.

[118]  Ingargiola, R. et al. (2020). A monocentric, open-label randomized standard-of-care controlled study of XONRID®, a medical device for the prevention and treatment of radiation-induced dermatitis in breast and head and neck cancer patients. *Radiation Oncology*. DOI: 10.1186/s13014-020-01633-0.

[119]  Chen, Y.-H. et al. (2020). Motor control integrated into muscle strengthening exercises has more effects on scapular muscle activities and joint range of motion before initiation of radiotherapy in oral cancer survivors with neck dissection: A randomized controlled trial. *PloS One*. DOI: 10.1371/journal.pone.0237133.

[120]  Sun, X.-S. et al. (2020). Debio 1143 and high-dose cisplatin chemoradiotherapy in high-risk locoregionally advanced squamous cell carcinoma of the head and neck: a double-blind, multicentre, randomised, phase 2 study. *The Lancet. Oncology*. DOI: 10.1016/S1470-2045(20)30327-2.

[121]  Held, T. et al. (2020). Carbon ion reirradiation compared to intensity-modulated re-radiotherapy for recurrent head and neck cancer (CARE): a randomized controlled trial. *Radiation Oncology*. DOI: 10.1186/s13014-020-01625-0.

[122]  Singh, N. et al. (2020). Comparison of extraoral and intraoral routes of glossopharyngeal nerve block for pain relief in patient with carcinoma tongue: A prospective randomized study. *Journal of Cancer Research and Therapeutics*. DOI: 10.4103/jcrt.JCRT_309_18.

[123]  Doppalapudi, R. et al. (2020). Effect of probiotic bacteria on oral Candida in head- and neck-radiotherapy patients: A randomized clinical trial. *Journal of Cancer Research and Therapeutics*. DOI: 10.4103/jcrt.JCRT_334_18.

[124]  Kristensen, M.B. et al. (2020). Effects of a Multidisciplinary Residential Nutritional Rehabilitation Program in Head and Neck Cancer Survivors-Results from the NUTRI-HAB Randomized Controlled Trial. *Nutrients*. DOI: 10.3390/nu12072117.

[125]  Palma, L.-F. et al. (2020). Leukocyte- and platelet-rich fibrin does not provide any additional benefit for tooth extraction in head and neck cancer patients post-radiotherapy: a randomized clinical trial. *Medicina Oral, Patologia Oral Y Cirugia Bucal*. DOI: 10.4317/medoral.23804.

[126]  da Silva, T.M.V. et al. (2021). Photobiomodulation for mucosal repair in patients submitted to dental extraction after head and neck radiation therapy: a double-blind randomized pilot study. *Supportive Care in Cancer: Official Journal of the Multinational Association of Supportive Care in Cancer*. DOI: 10.1007/s00520-020-05608-5.

[127]  Merlano, M.C. et al. (2020). Phase III Randomized Study of Induction Chemotherapy Followed by Definitive Radiotherapy + Cetuximab Versus Chemoradiotherapy in Squamous Cell Carcinoma of Head and Neck: The INTERCEPTOR-GONO Study (NCT00999700). *Oncology*. DOI: 10.1159/000507733.

[128]  Guglielmo, M. et al. (2020). A randomized, double-blind, placebo controlled, phase II study to evaluate the efficacy of ginseng in reducing fatigue in patients treated for head and neck cancer. *Journal of Cancer Research and Clinical Oncology*. DOI: 10.1007/s00432-020-03300-z.

[129]  Dymackova, R. et al. (2020). Acupuncture in the treatment of acute toxicity during and after head and neck cancer radiotherapy: Interim analysis of randomized prospective open-label trial. *Biomedical Papers of the Medical Faculty of the University Palacky, Olomouc, Czechoslovakia*. DOI: 10.5507/bp.2020.021.

[130]  Yen, C.-J. et al. (2020). Two-year follow-up of a randomized phase III clinical trial of nivolumab vs. the investigator’s choice of therapy in the Asian population for recurrent or metastatic squamous cell carcinoma of the head and neck (CheckMate 141). *Head & Neck*. DOI: 10.1002/hed.26331.

[131]  Louzeiro, G.C. et al. (2020). Effect of photobiomodulation on salivary flow and composition, xerostomia and quality of life of patients during head and neck radiotherapy in short term follow-up: A randomized controlled clinical trial. *Journal of Photochemistry and Photobiology. B, Biology*. DOI: 10.1016/j.jphotobiol.2020.111933.

[132] Li, T. et al. (2022). Effect of ‘timing it right’ on comprehensive unmet needs and psychological pain in patients with head and neck cancer undergoing radiotherapy: a randomized controlled trial. *Supportive Care in Cancer: Official Journal of the Multinational Association of Supportive Care in Cancer*. DOI: 10.1007/s00520-022-07072-9.

[133]  Riesterer, O. et al. (2020). Consolidation cetuximab after concurrent triplet radiochemotherapy+cetuximab in patients with advanced head and neck cancer: A randomized phase II study. *Radiotherapy and Oncology: Journal of the European Society for Therapeutic Radiology and Oncology*. DOI: 10.1016/j.radonc.2020.06.011.

[134]  Tao, Y. et al. (2020). Concurrent cisplatin and dose escalation with intensity-modulated radiotherapy (IMRT) versus conventional radiotherapy for locally advanced head and neck squamous cell carcinomas (HNSCC): GORTEC 2004-01 randomized phase III trial. *Radiotherapy and Oncology: Journal of the European Society for Therapeutic Radiology and Oncology*. DOI: 10.1016/j.radonc.2020.05.021.

[135]  Yan, J. et al. (2020). Mepitel Film is superior to Biafine cream in managing acute radiation-induced skin reactions in head and neck cancer patients: a randomised intra-patient controlled clinical trial. *Journal of Medical Radiation Sciences*. DOI: 10.1002/jmrs.397.

[136]  Rogers, S.N. et al. (2020). Improving quality of life through the routine use of the patient concerns inventory for head and neck cancer patients: baseline results in a cluster preference randomised controlled trial. *European archives of oto-rhino-laryngology: official journal of the European Federation of Oto-Rhino-Laryngological Societies (EUFOS): affiliated with the German Society for Oto-Rhino-Laryngology - Head and Neck Surgery*. DOI: 10.1007/s00405-020-06077-6.

[137]  Ridner, S.H. et al. (2021). Advanced pneumatic compression for treatment of lymphedema of the head and neck: a randomized wait-list controlled trial. *Supportive Care in Cancer: Official Journal of the Multinational Association of Supportive Care in Cancer*. DOI: 10.1007/s00520-020-05540-8.

[138]  Vesty, A. et al. (2020). Randomised, double-blind, placebo-controlled trial of oral probiotic Streptococcus salivarius M18 on head and neck cancer patients post-radiotherapy: a pilot study. *Scientific Reports*. DOI: 10.1038/s41598-020-70024-y.

[139]  Nakao, R. and Ueno, T. (2021). Effects of oral moisturizing gel containing propolis following head and neck radiotherapy: randomized controlled pilot trial. *BDJ Open*. DOI: 10.1038/s41405-021-00068-3.

**List of excluded articles after title/abstract analysis (with reasons for exclusion) (N = 131)**

***2020–2022:***

1. BMJ Open. 2022 Sep 14;12(9):e060912. doi: 10.1136/bmjopen-2022-060912.

Effect of melatonin on quality of life and symptoms in patients with cancer: a

systematic review and meta-analysis of randomised controlled trials.

Reason for exclusion: other study design

2. Front Oncol. 2022 Aug 24;12:935383. doi: 10.3389/fonc.2022.935383. eCollection

2022.

POD1UM-303/InterAACT 2: A phase III, global, randomized, double-blind study of

retifanlimab or placebo plus carboplatin-paclitaxel in patients with locally

advanced or metastatic squamous cell anal carcinoma.

Reason for exclusion: other area of cancer

3. Clin Neuropharmacol. 2022 Sep 2. doi: 10.1097/NCC.0000000000001149. Online ahead

of print.

Randomized Control Study of the Effects of Turmeric Mouthwash on Oral Health

Status, Treatment-Induced Mucositis, and Associated Oral Dysfunctions Among

Patients With Head and Neck Cancer.

Reason for exclusion: no full text available

4. Endocrinology. 2022 Sep 1:bqac139. doi: 10.1210/endocr/bqac139. Online ahead of

print.

Thyroid function and COVID-19 susceptibility and its severity: A Two-sample

Mendelian randomization study.

Reason for exclusion: other topic, not related to the research question

5. Trials. 2022 Aug 26;23(1):708. doi: 10.1186/s13063-022-06645-7.

Study protocol of brief intervention using gene polymorphism information for

excessive drinking among Japanese college students and adults aged 20-30 years:

a randomized controlled trial.

Reason for exclusion: other topic, not related to the research question

6. Eur J Cancer. 2022 Aug;171:242-258. doi: 10.1016/j.ejca.2022.04.038. Epub 2022

Jun 29.

Practice changing data and emerging concepts from recent radiation therapy

randomised clinical trials.

Reason for exclusion: overview of RCT

7. Int J Surg. 2022 Aug;104:106719. doi: 10.1016/j.ijsu.2022.106719. Epub 2022 Jun

25.

A commentary on "Endoscopic versus percutaneous biliary drainage for resectable

pancreatic head cancer with hyperbilirubinemia and impact on

pancreaticoduodenectomy: A randomized controlled study" (Int J Surg

2021;93:106043).

Reason for exclusion: other area of cancer

8. J Maxillofac Oral Surg. 2022 Jun;21(2):340-349. doi: 10.1007/s12663-021-01677-z.

Epub 2022 Jan 6.

Elective Neck Dissection Versus Therapeutic Neck Dissection in Clinically

Node-Negative Early Stage Oral Cancer: A Meta-analysis of Randomized Controlled

Trials.

Reason for exclusion: a meta-analysis of RCT’s

9. JAMA Otolaryngol Head Neck Surg. 2022 Jul 1;148(7):670-676. doi:

10.1001/jamaoto.2022.0890.

Comparison of Patients With Head and Neck Cancer in Randomized Clinical Trials

and Clinical Practice: A Systematic Review.

Reason for exclusion: no RCT

10. Integr Cancer Ther. 2022 Jan-Dec;21:15347354221098984. doi:

10.1177/15347354221098984.

Feasibility and Acceptability of a Multi-Modality Self-Management Intervention

for Head and Neck Cancer Caregivers: A Pilot Randomized Trial.

Reason for exclusion: not related to the research question - other topic

11. Cancers (Basel). 2022 Apr 24;14(9):2129. doi: 10.3390/cancers14092129.

Randomized Crossover Trial Evaluating Detoxification of Tobacco Carcinogens by

Broccoli Seed and Sprout Extract in Current Smokers.

Reason for exclusion: not related to the research question - other topic

12. Ann Oncol. 2022 Aug;33(8):804-813. doi: 10.1016/j.annonc.2022.04.074. Epub 2022

May 4.

Prognostic stratification of HPV-associated oropharyngeal cancer based on

CD103(+) immune cell abundance in patients treated on TROG 12.01 and De-ESCALaTE

randomized trials.

Reason for exclusion: secondary analysis of 2 RCT’s

13. J Clin Oncol. 2022 Jun 20;40(18):1967-1970. doi: 10.1200/JCO.22.00274. Epub 2022

Apr 21.

For Head and Neck Cancer, It Is Still Cisplatin, But How Much, How Often, and

How Tolerable? New Randomized Phase III Data For the Adjuvant Setting.

Reason for exclusion: no RCT

14. BMJ Open. 2022 Apr 4;12(4):e057128. doi: 10.1136/bmjopen-2021-057128.

Laparoscopic versus open pancreaticoduodenectomy for pancreatic ductal

adenocarcinoma: study protocol for a multicentre randomised controlled trial.

Reason for exclusion: study protocol – other type of cancer

15. Contemp Clin Trials Commun. 2022 Mar 20;27:100912. doi:

10.1016/j.conctc.2022.100912. eCollection 2022 Jun.

Preventive effect and safety of Chinese herbal medicine for oral mucositis

during radiotherapy in patients with head and neck cancer: Study protocol for a

randomized trial.

Reason for exclusion: no RCT - study protocol

16. Healthcare (Basel). 2022 Feb 26;10(3):442. doi: 10.3390/healthcare10030442.

Exercise for Trismus Prevention in Patients with Head and Neck Cancer: A Network

Meta-Analysis of Randomized Controlled Trials.

Reason for exclusion: no RCT - meta-analysis

17. Trials. 2022 Mar 18;23(1):221. doi: 10.1186/s13063-022-06163-6.

Efficacy of a new membrane obturator prosthesis in terms of speech, swallowing,

and the quality of life of patients with acquired soft palate defects: study

protocol of the VELOMEMBRANE randomized crossover trial.

Reason for exclusion: study protocol

18. Acta Otolaryngol. 2022 Feb;142(2):191-196. doi: 10.1080/00016489.2022.2035431.

Epub 2022 Feb 23.

Inflammatory Bowel disease promote oral cancer and pharyngeal cancer: new

evidence of Mendelian randomization.

Reason for exclusion: Mendelian randomization

19. Gulf J Oncolog. 2021 Sep;1(37):42-47.

Comparative Study of the Effect of Licorice Muco-adhesive Film on Radiotherapy

Induced Oral Mucositis, A Randomized Controlled Clinical Trial.

Reason for exclusion: no full text available

20. BMC Med. 2022 Jan 31;20(1):40. doi: 10.1186/s12916-022-02233-3.

Investigating the effect of sexual behaviour on oropharyngeal cancer risk: a

methodological assessment of Mendelian randomization.

Reason for exclusion: no RCT

21. Oral Oncol. 2022 Mar;126:105733. doi: 10.1016/j.oraloncology.2022.105733. Epub

2022 Jan 29.

Early relapse detection by monitoring of circulating cell-free DNA in patients

with localized head and neck squamous cell carcinoma: A subgroup analysis of the

multicenter randomized clinical trial IMSTAR-HN.

Reason for exclusion: secondary analysis of a RCT

22. J Clin Oncol. 2022 Mar 10;40(8):814-817. doi: 10.1200/JCO.21.02813. Epub 2022

Jan 25.

Interpreting ORATOR: Lessons Learned From a Randomized Comparison of Primary

Surgical and Radiation Approaches for Early-Stage Oropharyngeal Cancer.

Reason for exclusion: no RCT

23. BMJ Open. 2022 Jan 17;12(1):e056781. doi: 10.1136/bmjopen-2021-056781.

Preventive effects of betamethasone valerate ointment for radiation-induced

severe oral mucositis in patients with oral or oropharyngeal cancer: protocol

for a multicentre, phase II, randomised controlled trial (Bet-ROM study).

Reason for exclusion: protocol of a RCT

24. BMJ Open. 2022 Jan 11;12(1):e055814. doi: 10.1136/bmjopen-2021-055814.

Use of the ONCO-TreC electronic diary compared with a standard paper diary to

improve adherence to oral cancer therapy in patients with solid and

haematological tumours: protocol for a randomised controlled trial.

Reason for exclusion: protocol of a RCT

25. Laryngoscope. 2022 Aug;132(8):1609-1614. doi: 10.1002/lary.30006. Epub 2022 Jan

5.

Incidence of Head and Neck Cancer With Lung Cancer Screening: Secondary Analysis

of a Randomized Controlled Trial.

Reason for exclusion: other study design – secondary analysis of a RCT

26. Br J Cancer. 2022 Mar;126(5):831. doi: 10.1038/s41416-021-01678-2.

Correction to: Nationwide randomised trial evaluating elective neck dissection

for early stage oral cancer (SEND study) with meta-analysis and concurrent

real-world cohort.

Reason for exclusion: other study design - no RCT

27. Oral Oncol. 2022 Jan;124:105642. doi: 10.1016/j.oraloncology.2021.105642. Epub

2021 Nov 30.

Systematic review and meta-analysis of randomized controlled trials comparing

elective neck dissection versus sentinel lymph node biopsy in early-stage

clinically node-negative oral and/or oropharyngeal squamous cell carcinoma:

Evidence-base for practice and implications for research.

Reason for exclusion: other study design - no RCT

28. Radiother Oncol. 2022 Jan;166:137-144. doi: 10.1016/j.radonc.2021.11.021. Epub

2021 Nov 26.

Hyperbaric oxygen treatment of mandibular osteoradionecrosis: Combined data from

the two randomized clinical trials DAHANCA-21 and NWHHT2009-1.

Reason for exclusion: a combined data from two RCT’s

29. Int J Surg. 2021 Nov;95:106175. doi: 10.1016/j.ijsu.2021.106175. Epub 2021 Nov

6.

Author's response to Z. Huang on his commentary on "Effect of a perioperative

immune-enhancing diet in clean-contaminated head and neck cancer surgery: A

randomized controlled trial" (Int. J. Surg. 94 (2021) 106114).

Reason for exclusion: other study design - no RCT

30. Nat Med. 2021 Nov;27(11):1904-1909. doi: 10.1038/s41591-021-01562-9. Epub 2021

Nov 4.

Dalpiciclib or placebo plus fulvestrant in hormone receptor-positive and

HER2-negative advanced breast cancer: a randomized, phase 3 trial.

Reason for exclusion: other area of cancer

31. JAMA Otolaryngol Head Neck Surg. 2021 Dec 1;147(12):1071-1078. doi:

10.1001/jamaoto.2021.2776.

Incidence of Second Primary Lung Cancer After Low-Dose Computed Tomography vs

Chest Radiography Screening in Survivors of Head and Neck Cancer: A Secondary

Analysis of a Randomized Clinical Trial.

Reason for exclusion: a secondary analysis of a RCT

32. Int J Surg. 2021 Nov;95:106144. doi: 10.1016/j.ijsu.2021.106144. Epub 2021 Oct

21.

A commentary on "Endoscopic versus percutaneous biliary drainage for resectable

pancreatic head cancer with hyperbilirubinemia and impact on

pancreaticoduodenectomy: A randomized controlled study" [Int. J. Surg. 93 (2021

Sept 1) 106043].

Reason for exclusion: no RCT

33. Clin Nutr. 2021 Nov;40(11):5482-5485. doi: 10.1016/j.clnu.2021.09.040. Epub 2021

Oct 1.

Postoperative arginine-enriched immune modulating nutrition: Long-term survival

results from a randomised clinical trial in patients with oesophagogastric and

pancreaticobiliary cancer.

Reason for exclusion: other area of cancer

34. BMC Cancer. 2021 Oct 13;21(1):1100. doi: 10.1186/s12885-021-08826-0.

The PRO-ACTIVE trial protocol: a randomized study comparing the effectiveness of

PROphylACTic swallow InterVEntion for patients receiving radiotherapy for head

and neck cancer.

Reason for exclusion: study protocol - other study design - no RCT

35. Cancer Radiother. 2021 Dec;25(8):755-762. doi: 10.1016/j.canrad.2021.04.005.

Epub 2021 Sep 23.

A priori quality assurance using a benchmark case of the randomized phase 2

GORTEC 2014-14 in oligometastatic head and neck cancer patients.

Reason for exclusion: other study design - no RCT

36. Int J Surg. 2021 Oct;94:106114. doi: 10.1016/j.ijsu.2021.106114. Epub 2021 Sep

20.

Comment on "Effect of a perioperative immune-enhancing diet in

clean-contaminated head and neck cancer surgery: A randomized controlled trial"

(Int J Surg 2021; 93:106051).

Reason for exclusion: no RCT

37. Ther Adv Med Oncol. 2021 Sep 11;13:17588359211025872. doi:

10.1177/17588359211025872. eCollection 2021.

The efficacy of immunonutrition in improving tolerance to chemoradiotherapy in

patients with head and neck cancer, receiving nutritional counseling: study

protocol of a randomized, open-label, parallel group, bicentric pilot study.

Reason for exclusion: only study protocol of a RCT

38. Photodiagnosis Photodyn Ther. 2021 Dec;36:102523. doi:

10.1016/j.pdpdt.2021.102523. Epub 2021 Sep 4.

Ablative fractional laser-assisted photodynamic therapy vs. ablative fractional

laser for oral leukoplakia treatment: A randomized, controlled pilot study.

Reason for exclusion: not related to the research question

39. Perioper Med (Lond). 2021 Aug 19;10(1):28. doi: 10.1186/s13741-021-00195-3.

The efficacy of computer-assisted cognitive behavioral therapy (cCBT) on

psychobiological responses and perioperative outcomes in patients undergoing

functional endoscopic sinus surgery: a randomized controlled trial.

Reason for exclusion: not related to the research question

40. J Indian Prosthodont Soc. 2021 Jul-Sep;21(3):249-255. doi:

10.4103/jips.jips_98_21.

Speech intelligibility, nasal resonance, and swallowing ability of maxillectomy

patients with customized obturator: A non randomized controlled study.

Reason for exclusion: no RCT

41. Int J Surg. 2021 Sep;93:106043. doi: 10.1016/j.ijsu.2021.106043. Epub 2021 Aug

8.

Endoscopic versus percutaneous biliary drainage for resectable pancreatic head

cancer with hyperbilirubinemia and impact on pancreaticoduodenectomy: A

randomized controlled study.

Reason for exclusion: other area of cancer

42. BMC Cancer. 2021 Jul 15;21(1):812. doi: 10.1186/s12885-021-08473-5.

Adenoid cystic Carcinoma and Carbon ion Only irradiation (ACCO): Study protocol

for a prospective, open, randomized, two-armed, phase II study.

Reason for exclusion: study protocol

43. Trials. 2021 Jul 5;22(1):428. doi: 10.1186/s13063-021-05373-8.

SAVER: sodium valproate for the epigenetic reprogramming of high-risk oral

epithelial dysplasia-a phase II randomised control trial study protocol.

Reason for exclusion: study protocol

44. BMJ Open. 2021 Jun 21;11(6):e045741. doi: 10.1136/bmjopen-2020-045741.

Getting Recovery Right After Neck Dissection (GRRAND-F): mixed-methods

feasibility study to design a pragmatic randomised controlled trial protocol.

Reason for exclusion: no RCT

45. Int J Cancer. 2021 Oct 15;149(8):1619-1620. doi: 10.1002/ijc.33712. Epub 2021

Jun 30.

Outcome of the randomized control screening trials on oral, cervix and breast

cancer from India and way forward in COVID-19 pandemic situation.

Reason for exclusion: no RCT

46. Dysphagia. 2022 Aug;37(4):749-762. doi: 10.1007/s00455-021-10320-5. Epub 2021

Jun 11.

Swallowing Exercise During Head and Neck Cancer Treatment: Results of a

Randomized Trial.

Reason for exclusion: no RCT

47. Contemp Clin Trials. 2021 Aug;107:106448. doi: 10.1016/j.cct.2021.106448. Epub

2021 May 21.

The Head and Neck Survivorship Tool (HN-STAR) Trial (WF-1805CD): A protocol for

a cluster-randomized, hybrid effectiveness-implementation, pragmatic trial to

improve the follow-up care of head and neck cancer survivors.

Reason for exclusion: no RCT

48. J Egypt Natl Canc Inst. 2021 May 22;33(1):12. doi: 10.1186/s43046-021-00069-1.

Post hoc analysis of a randomized controlled trial comparing concurrent

chemoradiation with cisplatin versus nimotuzumab-cisplatin, focusing on acute

oral mucositis.

Reason for exclusion: analysis of a RCT

49. Integr Cancer Ther. 2021 Jan-Dec;20:15347354211006474. doi:

10.1177/15347354211006474.

Interventions for Trismus in Head and Neck Cancer Patients: A Systematic Review

of Randomized Controlled Trials.

Reason for exclusion: a systematic review of RCT’s

50. Food Funct. 2021 Apr 21;12(8):3352-3365. doi: 10.1039/d0fo02808h. Epub 2021 Mar

31.

Role of honey in preventing radiation-induced oral mucositis: a meta-analysis of

randomized controlled trials.

Reason for exclusion: a meta-analysis of RCT’s

51. PLoS Genet. 2021 Apr 22;17(4):e1009525. doi: 10.1371/journal.pgen.1009525.

eCollection 2021 Apr.

Using genetic variants to evaluate the causal effect of cholesterol lowering on

head and neck cancer risk: A Mendelian randomization study.

Reason for exclusion: mendelian randomization study

52. Asian Pac J Cancer Prev. 2021 Mar 1;22(3):711-717. doi:

10.31557/APJCP.2021.22.3.711.

Comparison of Interventional Methods to Motivate and Change the Behavioural

Stage of Smokers to Quit Smoking- A Hospital Based Randomised Controlled Trial.

Reason for exclusion: not related to the research question

53. BMC Palliat Care. 2021 Mar 19;20(1):45. doi: 10.1186/s12904-021-00735-0.

Opioid therapy vs. multimodal analgesia in head and neck Cancer (OPTIMAL-HN):

study protocol for a randomized clinical trial.

Reason for exclusion: study protocol

54. Med Oncol. 2021 Mar 8;38(4):35. doi: 10.1007/s12032-021-01479-w.

nab-Paclitaxel and cisplatin followed by cisplatin and radiation (Arm 1) and

nab-paclitaxel followed by cetuximab and radiation (Arm 2) for locally advanced

head and neck squamous-cell carcinoma: a multicenter, non-randomized phase 2

trial.

Reason for exclusion: no RCT

55. Radiother Oncol. 2021 May;158:326. doi: 10.1016/j.radonc.2021.02.023. Epub 2021

Feb 26.

Response to the letter to the editor "Exercise therapy for cancer

treatment-induced trismus in patients with head and neck cancer: A systematic

review and meta-analysis of randomized controlled trials regards to Chiu et

al.".

Reason for exclusion: no RCT– systematic review and meta-analysis

56. Radiother Oncol. 2021 May;158:325. doi: 10.1016/j.radonc.2021.02.025. Epub 2021

Feb 26.

Letter to the editor regarding Shao et al., exercise therapy for cancer

treatment-induced trismus in patients with head and neck cancer: A systematic

review and meta-analysis of randomized controlled trials.

Reason for exclusion: no RCT – systematic review and meta-analysis

57. Eur Arch Otorhinolaryngol. 2021 Nov;278(11):4125-4133. doi:

10.1007/s00405-021-06694-9. Epub 2021 Feb 19.

Reporting quality of surgical randomised controlled trials in head and neck

cancer: a systematic review.

Reason for exclusion: no RCT – systematic review and meta-analysis

58. Int J Oral Maxillofac Surg. 2021 Nov;50(11):1403-1407. doi:

10.1016/j.ijom.2021.01.010. Epub 2021 Feb 16.

Prognostic value of elective neck dissection in adenoid cystic carcinoma of head

and neck: a meta-analysis: A call for randomized trials and international

consensus.

Reason for exclusion: no RCT – a meta-analysis

59. Ann Gastroenterol Surg. 2020 Sep 15;5(1):111-118. doi: 10.1002/ags3.12399.

eCollection 2021 Jan.

Multicenter randomized phase II trial of prophylactic right-half dissection of

superior mesenteric artery nerve plexus in pancreatoduodenectomy for pancreatic

head cancer.

Reason for exclusion: other area of cancer

60. Medicine (Baltimore). 2021 Jan 29;100(4):e24339. doi:

10.1097/MD.0000000000024339.

Anti-PD1/PD-L1 monotherapy vs standard of care in patients with recurrent or

metastatic head and neck squamous cell carcinoma: A meta-analysis of randomized

controlled trials.

Reason for exclusion: no RCT – meta-analysis

61. Radiother Oncol. 2021 Mar;156:281-293. doi: 10.1016/j.radonc.2021.01.013. Epub

2021 Jan 27.

Meta-analysis of chemotherapy in head and neck cancer (MACH-NC): An update on

107 randomized trials and 19,805 patients, on behalf of MACH-NC Group.

Reason for exclusion: no RCT – meta-analysis

62. Anesth Essays Res. 2020 Apr-Jun;14(2):355. doi: 10.4103/0259-1162.297841.

Retraction: Does the preoperative administration of tranexamic acid reduce

perioperative blood loss and transfusion requirements after head neck cancer

surgery? A randomized, controlled trial.

Reason for exclusion: retracted article

63. Trials. 2021 Jan 8;22(1):40. doi: 10.1186/s13063-020-04933-8.

Evaluation of robotic versus open partial pancreatoduodenectomy-study protocol

for a randomised controlled pilot trial (EUROPA, DRKS00020407).

Reason for exclusion: other area of cancer and study protocol

64. J Dent Sci. 2021 Jan;16(1):404-409. doi: 10.1016/j.jds.2020.08.005. Epub 2020

Aug 26.

Semi-fixed versus fixed oral appliance therapy for obstructive sleep apnea: A

randomized crossover pilot study.

Reason for exclusion: not related to the research question

65. BMJ Open. 2020 Dec 18;10(12):e038867. doi: 10.1136/bmjopen-2020-038867.

Cost-effectiveness of pembrolizumab for treatment of platinum-resistant

recurrent or metastatic head and neck squamous cell carcinoma in China: an

economic analysis based on a randomised, open-label, phase III trial.

Reason for exclusion: no RCT

66. Int J Nurs Stud. 2021 Feb;114:103827. doi: 10.1016/j.ijnurstu.2020.103827. Epub

2020 Nov 11.

Swallowing exercises for head and neck cancer patients: A systematic review and

meta-analysis of randomized control trials.

Reason for exclusion: no RCT – systematic review and meta-analysis

67. Ther Adv Med Oncol. 2020 Dec 8;12:1758835920975355. doi:

10.1177/1758835920975355. eCollection 2020.

Comparative efficacy and safety of radiotherapy/cetuximab versus

radiotherapy/chemotherapy for locally advanced head and neck squamous cell

carcinoma patients: a systematic review of published, primarily non-randomized,

data.

Reason for exclusion: no RCT – systematic review

68. Medicine (Baltimore). 2020 Dec 11;99(50):e23080. doi:

10.1097/MD.0000000000023080.

Comparison of the efficacy of the anterolateral thigh flap for perforator

localization in the repair of head and neck soft tissue defects patients: A

protocol study for a randomized controlled trial.

Reason for exclusion: no RCT – study protocol

69. BMC Cancer. 2020 Dec 14;20(1):1219. doi: 10.1186/s12885-020-07709-0.

Application of Random Forest and data integration identifies three dysregulated

genes and enrichment of Central Carbon Metabolism pathway in Oral Cancer.

Reason for exclusion: not related to the research question

70. JMIR Res Protoc. 2020 Dec 9;9(12):e21693. doi: 10.2196/21693.

App-Controlled Treatment Monitoring and Support for Head and Neck Cancer

Patients (APCOT): Protocol for a Prospective Randomized Controlled Trial.

Reason for exclusion: Protocol for a RCT

71. Nat Commun. 2020 Nov 27;11(1):6071. doi: 10.1038/s41467-020-19822-6.

A multivariable Mendelian randomization analysis investigating smoking and

alcohol consumption in oral and oropharyngeal cancer.

Reason for exclusion: no RCT – mendelian randomization

72. Chin Med J (Engl). 2020 Nov 18;134(4):489-491. doi:

10.1097/CM9.0000000000001231.

Comparative role of real-world study and traditional randomized controlled

trials in head and neck cancer: a literature-based analysis.

Reason for exclusion: no RCT

73. BMJ Open. 2020 Sep 29;10(9):e038606. doi: 10.1136/bmjopen-2020-038606.

Evaluation of the efficacy of low concentration fluoride gel using custom trays

to prevent radiation-related dental caries in patients with head and neck

cancer: protocol for a randomised controlled phase III trial (FluCar study).

Reason for exclusion: protocol for a RCT

74. Langenbecks Arch Surg. 2021 May;406(3):547-561. doi: 10.1007/s00423-020-01999-5.

Epub 2020 Sep 26.

Meta-analysis and trial sequential analysis of randomised controlled trials

comparing standard versus extended lymphadenectomy in pancreatoduodenectomy for

adenocarcinoma of the head of pancreas.

Reason for exclusion: other area of cancer + meta-analysis

75. Cancer Prev Res (Phila). 2021 Feb;14(2):275-284. doi:

10.1158/1940-6207.CAPR-20-0234. Epub 2020 Sep 21.

A Randomized Controlled Trial on Efficacy of Surgical Excision of Nondysplastic

Leukoplakia to Prevent Oral Cancer.

Reason for exclusion: not related to the research question

76. Radiother Oncol. 2020 Oct;151:249-255. doi: 10.1016/j.radonc.2020.08.024. Epub

2020 Sep 3.

Exercise therapy for cancer treatment-induced trismus in patients with head and

neck cancer: A systematic review and meta-analysis of randomized controlled

trials.

Reason for exclusion: a systematic review and meta-analysis

77. J Cancer Res Clin Oncol. 2021 Feb;147(2):539-547. doi:

10.1007/s00432-020-03347-y. Epub 2020 Aug 14.

Effects of N-acetylcysteine inhalation therapy on the quality of life of

patients with head and neck cancer who are receiving radiation therapy: a

prospective non-randomized controlled multi-center study.

Reason for exclusion: no RCT

78. Radiat Oncol. 2020 Aug 14;15(1):196. doi: 10.1186/s13014-020-01636-x.

Preservation of swallowing in resected oral cavity squamous cell carcinoma:

examining radiation volume effects (PRESERVE): study protocol for a randomized

phase II trial.

Reason for exclusion: study protocol

79. Cancer Manag Res. 2020 Jul 3;12:5353-5363. doi: 10.2147/CMAR.S251021.

eCollection 2020.

Signatures of Mucosal Microbiome in Oral Squamous Cell Carcinoma Identified

Using a Random Forest Model.

Reason for exclusion: not related to the research question

80. Int J Surg Protoc. 2020 Jul 15;23:1-5. doi: 10.1016/j.isjp.2020.07.001.

eCollection 2020.

Oral cancer screening using mobile phone-based(mHealth) approach versus

conventional oral examination approach, protocol of a cluster randomized study

with cost-effectiveness analysis.

Reason for exclusion: study protocol

81. J Hepatobiliary Pancreat Sci. 2020 Oct;27(10):690-699. doi: 10.1002/jhbp.811.

Epub 2020 Aug 16.

A multicenter prospective randomized controlled trial for preoperative biliary

drainage with uncovered metal versus plastic stents for resectable periampullary

cancer.

Reason for exclusion: other area of cancer

82. PLoS Med. 2020 Jul 23;17(7):e1003178. doi: 10.1371/journal.pmed.1003178.

eCollection 2020 Jul.

Smoking, alcohol consumption, and cancer: A mendelian randomisation study in UK

Biobank and international genetic consortia participants.

Reason for exclusion: a Mendelian randomization

83. J Contemp Dent Pract. 2020 May 1;21(5):521-531.

Chemopreventive Synergism between Green Tea Extract and Curcumin in Patients

with Potentially Malignant Oral Disorders: A Double-blind, Randomized

Preliminary Study.

Reason for exclusion: not related to the research question

84. Br J Cancer. 2020 Sep;123(7):1200-1201. doi: 10.1038/s41416-020-0983-7. Epub

2020 Jul 16.

Reply to Comment(s) on "Nationwide randomised trial evaluating elective neck

dissection for early stage oral cancer (SEND study) with meta-analysis and

concurrent real-world cohort".

Reason for exclusion: no RCT

85. Br J Cancer. 2020 Sep;123(7):1198-1199. doi: 10.1038/s41416-020-0981-9. Epub

2020 Jul 16.

Comment on "Nationwide randomised trial evaluating elective neck dissection for

early-stage oral cancer (SEND study) with meta-analysis and concurrent

real-world cohort.".

Reason for exclusion: no RCT

86. Radiother Oncol. 2020 Aug;149:246. doi: 10.1016/j.radonc.2020.06.026. Epub 2020

Jun 27.

Challenges in conducting large randomised control trials of palliative

radiotherapy in head and neck cancer.

Reason for exclusion: no RCT

87. Tob Prev Cessat. 2020 Jan 15;6:4. doi: 10.18332/tpc/115028. eCollection 2020.

Brief Intervention for Tobacco when Diagnosed with Oral Cancer (BITDOC): Study

protocol of a randomized clinical trial studying efficacy of brief tobacco

cessation intervention, Chhattisgarh, India.

Reason for exclusion: study protocol

88. Int J Radiat Oncol Biol Phys. 2020 Jul 1;107(3):606. doi:

10.1016/j.ijrobp.2020.03.010.

Erratum to: Britton B, Baker AL, Wolfenden L, Wratten C, Bauer J, Beck AK,

McCarter K, Harrowfield J, Isenring E, Tang C, Oldmeadow C, and Carter G. Eating

As Treatment (EAT): A Stepped-Wedge, Randomized Controlled Trial of a Health

Behavior Change Intervention Provided by Dietitians to Improve Nutrition in

Patients With Head and Neck Cancer Undergoing Radiation Therapy (TROG 12.03).

Int J Radiat Oncol Biol Phys 2019.

Reason for exclusion: no RCT

89. Nutr Cancer. 2021;73(5):740-749. doi: 10.1080/01635581.2020.1776884. Epub 2020

Jun 9.

Prophylactic and Therapeutic Effects of Curcumin on Treatment-Induced Oral

Mucositis in Patients with Head and Neck Cancer: A Meta-Analysis of Randomized

Controlled Trials.

Reason for exclusion: a meta-analysis of RCT‘s

90. Complement Ther Med. 2020 Jun;51:102397. doi: 10.1016/j.ctim.2020.102397. Epub

2020 Apr 30.

Efficacy of the plantago major L. syrup on radiation induced oral mucositis in

head and neck cancer patients: A randomized, double blind, placebo-controlled

clinical trial.

Reason for exclusion: different time

91. Trials. 2020 May 25;21(1):424. doi: 10.1186/s13063-020-04307-0.

Radiotherapy-related skin toxicity (RAREST-02): A randomized trial testing the

effect of a mobile application reminding head-and-neck cancer patients to

perform skin care (reminder app) on radiation dermatitis.

Reason for exclusion: different time

***2010–2012:***

1. Radiother Oncol. 2012 Aug;104(2):213-8. doi: 10.1016/j.radonc.2012.05.004. Epub

2012 Jun 21.

Predictors of severe late radiotherapy-related toxicity after hyperfractionated

radiotherapy with or without concomitant cisplatin in locally advanced head and

neck cancer. Secondary retrospective analysis of a randomized phase III trial

(SAKK 10/94).

Reason for exclusion: secondary analysis

2. J Otolaryngol Head Neck Surg. 2012 Apr;41 Suppl 1:S71-4.

Effect of intraoperative dopamine on free flap survival in a rat model: a

double-blind, randomized, controlled trial.

Reason for exclusion: no patients , only rats

3. Dig Endosc. 2012 May;24(3):168-74. doi: 10.1111/j.1443-1661.2011.01180.x. Epub

2011 Jul 13.

Prospective randomized controlled study comparing cell block method and

conventional smear method for pancreatic juice cytology.

Reason for exclusion: other area of cancer

4. Radiother Oncol. 2012 Apr;103(1):82-7. doi: 10.1016/j.radonc.2012.02.006. Epub

2012 Mar 21.

Novel approaches to improve the therapeutic index of head and neck radiotherapy:

an analysis of data from the PARSPORT randomised phase III trial.

Reason for exclusion: no RCT

5. Cancer. 2012 Oct 1;118(19):4694-705. doi: 10.1002/cncr.27449. Epub 2012 Mar 20.

Pemetrexed in combination with cisplatin versus cisplatin monotherapy in

patients with recurrent or metastatic head and neck cancer: final results of a

randomized, double-blind, placebo-controlled, phase 3 study.

Reason for exclusion: different time

6. Contemp Clin Trials. 2012 Jul;33(4):700-11. doi: 10.1016/j.cct.2012.02.017. Epub

2012 Mar 2.

Acupuncture for dysphagia after chemoradiation in head and neck cancer:

rationale and design of a randomized, sham-controlled trial.

Reason for exclusion: no RCT

7. J Cancer Res Ther. 2012 Jan;8 Suppl 1:S94-9. doi: 10.4103/0973-1482.92221.

Prospective analysis of reasons for non-enrollment in a phase III randomized

controlled trial.

Reason for exclusion: not related to the research question

8. Eur J Cancer Prev. 2012 Sep;21(5):460-6. doi: 10.1097/CEJ.0b013e32834fdb6d.

Clinical evaluation of an autofluorescence diagnostic device for oral cancer

detection: a prospective randomized diagnostic study.

Reason for exclusion: other topic: diagnostic instead of therapy

9. J Hepatobiliary Pancreat Sci. 2012 May;19(3):230-41. doi:

10.1007/s00534-011-0466-6.

Standard versus extended lymphadenectomy in radical pancreatoduodenectomy for

ductal adenocarcinoma of the head of the pancreas: long-term results of a

Japanese multicenter randomized controlled trial.

Reason for exclusion: other area of cancer

10. BMC Cancer. 2011 Oct 25;11:462. doi: 10.1186/1471-2407-11-462.

Canadian Optically-guided approach for Oral Lesions Surgical (COOLS) trial:

study protocol for a randomized controlled trial.

Reason for exclusion: study protocol

11. Dig Endosc. 2011 Oct;23(4):310-5. doi: 10.1111/j.1443-1661.2011.01124.x. Epub

2011 Apr 4.

Results of a Japanese multicenter, randomized trial of endoscopic stenting for

non-resectable pancreatic head cancer (JM-test): Covered Wallstent versus

DoubleLayer stent.

Reason for exclusion: other area of cancer

12. Stat Med. 2011 Oct 30;30(24):2890-910. doi: 10.1002/sim.4331. Epub 2011 Aug 8.

Optimal response-adaptive randomized designs for multi-armed survival trials.

Reason for exclusion: no RCT

13. Cancer Prev Res (Phila). 2011 Jul;4(7):994-1001. doi:

10.1158/1940-6207.CAPR-10-0301.

A DNA vaccine against ERBB2 impairs chemical carcinogenesis in random-bred

hamsters.

Reason for exclusion: no RCT

14. Oral Oncol. 2011 May;47(5):320-4. doi: 10.1016/j.oraloncology.2011.03.009. Epub

2011 Apr 2.

A meta-analysis of the randomized controlled trials on elective neck dissection

versus therapeutic neck dissection in oral cavity cancers with clinically

node-negative neck.

Reason for exclusion: a meta-analysis

15. Dig Endosc. 2011 Apr;23(2):135-9. doi: 10.1111/j.1443-1661.2010.01051.x. Epub

2010 Dec 7.

Clinical trial: a randomized study comparing the durability of silicone and

latex percutaneous endoscopic gastrostomy tubes.

Reason for exclusion: not related to the research question

16. Br J Oral Maxillofac Surg. 2011 Jul;49(5):412-3. doi:

10.1016/j.bjoms.2011.01.007. Epub 2011 Feb 25.

Active versus passive neck drainage in head and neck oncology surgery:

completing the re-audit cycle: Re: Batstone MD, Lowe D, Shaw RJ, Brown JS,

Vaughan ED, Rogers SN. Passive versus active drainage following neck dissection:

a non-randomised prospective study.

Reason for exclusion: no RCT

17. Head Neck. 2012 Jan;34(1):50-8. doi: 10.1002/hed.21698. Epub 2011 Feb 14.

Novel head and neck cancer survival analysis approach: random survival forests

versus Cox proportional hazards regression.

Reason for exclusion: not related to the research question

18. J Clin Oncol. 2010 Oct 10;28(29):4450-6. doi: 10.1200/JCO.2010.30.3446. Epub

2010 Sep 13.

Adjuvant gemcitabine alone versus gemcitabine-based chemoradiotherapy after

curative resection for pancreatic cancer: a randomized

EORTC-40013-22012/FFCD-9203/GERCOR phase II study.

Reason for exclusion: other area of cancer

19. Clin Oncol (R Coll Radiol). 2011 Feb;23(1):29-33. doi:

10.1016/j.clon.2010.08.007. Epub 2010 Sep 9.

Correlation of currently used radiobiological parameters with local control and

acute and late mucosal toxicity in randomised studies of altered fractionation

for locally advanced head and neck cancer.

Reason for exclusion: a meta-analysis

20. Nutr Cancer. 2010;62(5):682-91. doi: 10.1080/01635581003605532.

Discrepancy of the effects of zinc supplementation on the prevention of

radiotherapy-induced mucositis between patients with nasopharyngeal carcinoma

and those with oral cancers: subgroup analysis of a double-blind, randomized

study.

Reason for exclusion. Subgroup analysis, secondary analysis

21. Cardiovasc Intervent Radiol. 2011 Apr;34(2):352-61. doi:

10.1007/s00270-010-9880-4. Epub 2010 May 14.

Percutaneous palliation of pancreatic head cancer: randomized comparison of

ePTFE/FEP-covered versus uncovered nitinol biliary stents.

Reason for exclusion: other area of cancer

22. Otolaryngol Head Neck Surg. 2010 Mar;142(3):306-9. doi:

10.1016/j.otohns.2009.11.011.

Diagnostic efficacy of surgeon-performed ultrasound-guided fine needle

aspiration: a randomized controlled trial.

Reason for exclusion: not related to the research question

***2000–2002:***

1. J Clin Oncol. 2002 Oct 1;20(19):3956-63. doi: 10.1200/JCO.2002.05.046.

Prophylaxis of radiation-associated mucositis in conventionally treated patients

with head and neck cancer: a double-blind, phase III, randomized, controlled

trial evaluating the clinical efficacy of an antimicrobial lozenge using a

validated mucositis scoring system.

Reason for exclusion: different time

2. Int J Radiat Oncol Biol Phys. 2002 Oct 1;54(2):479-85. doi:

10.1016/s0360-3016(02)02935-8.

Comparison of granulocyte-macrophage colony-stimulating factor and sucralfate

mouthwashes in the prevention of radiation-induced mucositis: a double-blind

prospective randomized phase III study.

Reason for exclusion: different time

3. Int J Radiat Oncol Biol Phys. 2002 Sep 1;54(1):300. doi:

10.1016/s0360-3016(02)02909-7.

In regard to Staar et al.: Intensified hyperfractionated accelerated

radiotherapy limits the additional benefits of simultaneous chemotherapy-results

of a multicentric randomized German trial in advanced head-and-neck cancer.

IJROBP 2001;50:1161-1171.

Reason for exclusion: no RCT

4. Gan To Kagaku Ryoho. 2002 Jun;29(6):911-6.

[Non-randomized clinical study comparing chemotherapy plus radiotherapy with

radiotherapy alone in neoadjuvant therapy for oral cancer].

Reason for exclusion: no RCT

5. Ann Oncol. 2001 Nov;12(11):1595-9. doi: 10.1023/a:1013185402896.

Result of two randomized trials comparing nolatrexed (Thymitaq) versus

methotrexate in patients with recurrent head and neck cancer.

Reason for exclusion: report on two RCT‘s

6. Otolaryngol Pol. 2001;55(3):287-92.

[The quality of life in head and neck cancer patients: description of randomized

examination formula based on standardized questionnaires EORTC QLQ C-30, EORTC

QTQ-H-N35 and Kiel Questionnaire].

Reason for exclusion: not related to the research question + article in polish

7. Strahlenther Onkol. 2001 Sep;177(9):495-6. doi: 10.1007/BF03353388.

[Effects of an adjuvant mistletoe therapy in patients with surgically treated

head and neck neoplasms: a randomized clinical trial].

Reason for exclusion: article in german

8. Int J Radiat Oncol Biol Phys. 2001 Oct 1;51(2):563. doi:

10.1016/s0360-3016(01)01661-3.

A Radiation Therapy Oncology Group (RTOG) phase III randomized study to compare

hyperfractionation and two variants of accelerated fractionation to

standard-fractionation radiotherapy for head-and-neck squamous cell carcinomas:

first report of RTOG 9003: in regard to Fu et al. IJROBP 2000;48:7-16. Actuarial

estimates of late normal-tissue effects...now!

Reason for exclusion: a report of RTOG – no RCT

9. Oncology. 2001;61(3):197-204. doi: 10.1159/000055375.

Clinical prognostic factors for patients with recurrent head and neck cancer:

implications for randomized trials.

Reason for exclusion: no RCT

10. Oral Oncol. 2001 Oct;37(7):548-52. doi: 10.1016/s1368-8375(01)00017-3.

Randomised trial of the psychological effect of information about oral cancer in

primary care settings.

Reason for exclusion: not related to the research question

11. Lancet. 2001 Aug 4;358(9279):375-81. doi: 10.1016/S0140-6736(01)05558-1.

Monitoring of large randomised clinical trials: a new approach with Bayesian

methods.

Reason for exclusion: not related to the research question

12. Cancer Radiother. 2001 Apr;5(2):207-8. doi: 10.1016/s1278-3218(00)00078-0.

Randomized clinical trial on 7-day continuous accelerated irradiation (CAIR) of

head and neck cancer. Report on 3-year tumour control and normal tissue

toxicity.

Reason for exclusion: no RCT

13. Cancer Invest. 2001;19(2):107-13. doi: 10.1081/cnv-100000145.

Economic analysis of amifostine as adjunctive support for patients with advanced

head and neck cancer: preliminary results from a randomized phase II clinical

trial from Germany.

Reason for exclusion: no RCT

14. Int J Radiat Oncol Biol Phys. 2001 Mar 15;49(4):917-30. doi:

10.1016/s0360-3016(00)01456-5.

Prophylaxis of oral mucositis in irradiated head-and-neck cancer patients: a

proposed classification scheme of interventions and meta-analysis of randomized

controlled trials.

Reason for exclusion: Meta-analysis of RCT‘s

15. J Clin Oncol. 2001 Feb 15;19(4):1233-4. doi: 10.1200/JCO.2001.19.4.1233.

Phase III randomized trial of amifostine as a radioprotector in head and neck

cancer.

Reason for exclusion: a comment – no RCT

16. Oral Oncol. 2001 Jan;37(1):99-102. doi: 10.1016/s1368-8375(00)00069-5.

Immediate knowledge increase from an oral cancer information leaflet in patients

attending a primary health care facility: a randomised controlled trial.

Reason for exclusion: not related to the research question

17. Orv Hetil. 2000 Nov 5;141(45):2433-7.

[Randomized study of cisplatin-based combination chemotherapy for the treatment

of planocellular cancer of the head and neck region].

Reason for exclusion: article in Hungarian

18. Gan To Kagaku Ryoho. 2000 Sep;27(10):1557-63.

[A randomized crossover comparison of azasetron alone and azasetron plus

dexamethasone for the prevention of nausea and vomiting by chemotherapy

including cisplatin].

Reason for exclusion: article in Japanese

**List of excluded articles after full-text analysis (with reasons for exclusion) (N = 12)**

***2020–2022:***

1. PLoS One. 2022 May 10;17(5):e0267887. doi: 10.1371/journal.pone.0267887.

eCollection 2022.

Acceptance and commitment therapy versus mindfulness-based stress reduction for

newly diagnosed head and neck cancer patients: A randomized controlled trial

assessing efficacy for positive psychology, depression, anxiety, and quality of

life.

Reason for exclusion: study protocol

2. Front Oncol. 2021 Jun 7;11:650335. doi: 10.3389/fonc.2021.650335. eCollection

2021.

Determining Clinical Patient Selection Guidelines for Head and Neck Adaptive

Radiation Therapy Using Random Forest Modelling and a Novel Simplification

Heuristic.

Reason for exclusion: other topic

3. Strahlenther Onkol. 2020 Nov;196(11):1062-1064. doi: 10.1007/s00066-020-01672-2.

[Randomized controlled trial for palliative radiotherapy of head and neck

cancer-challenges remain].

Reason for exclusion: [Article in German]

4. Eur Arch Otorhinolaryngol. 2021 Sep;278(9):3435-3449. doi:

10.1007/s00405-020-06533-3. Epub 2020 Dec 21.

Improving quality of life through the routine use of the patient concerns

inventory for head and neck cancer patients: main results of a cluster

preference randomised controlled trial.

Reason for exclusion: Similar to the article from 2020

5. Clin Oral Investig. 2021 Apr;25(4):1815-1827. doi: 10.1007/s00784-020-03484-1.

Epub 2020 Aug 10.

Efficacy of gel-based artificial saliva on Candida colonization and saliva

properties in xerostomic post-radiotherapy head and neck cancer patients: a

randomized controlled trial.

Reason for exclusion: secondary analysis of a RCT

6. Nutrients. 2020 Aug 4;12(8):2332. doi: 10.3390/nu12082332.

Assessing Adherence, Competence and Differentiation in a Stepped-Wedge

Randomised Clinical Trial of a Complex Behaviour Change Intervention.

Reason for exclusion: other topic

7. Strahlenther Onkol. 2020 Sep;196(9):834-836. doi: 10.1007/s00066-020-01639-3.

[A superoxide dismutase mimetic (GC4419) vs. placebo to reduce severe oral

mucositis due to concurrent radiotherapy and cisplatin for head and neck cancer:

a phase-IIb randomized double-blind study].

Reason for exclusion: Article in German

***2010–2012:***

1. Acta Oncol. 2011 Oct;50(7):1006-14. doi: 10.3109/0284186X.2011.592650. Epub 2011

Jul 26.

Does transfusion improve the outcome for HNSCC patients treated with

radiotherapy? - results from the randomized DAHANCA 5 and 7 trials.

Reason for exclusion: no RCT

2. J Otolaryngol Head Neck Surg. 2011 Feb;40 Suppl 1:S59-64.

Heparin versus tirofiban in microvascular anastomosis: randomized controlled

trial in a rat model.

Reason for exclusion: no patients

3. Radiother Oncol. 2011 Jul;100(1):49-55. doi: 10.1016/j.radonc.2011.02.010. Epub

2011 Mar 21.

The influence of HPV-associated p16-expression on accelerated fractionated

radiotherapy in head and neck cancer: evaluation of the randomised DAHANCA 6&7

trial.

Reason for exclusion: no RCT

4. J Clin Oncol. 2010 Mar 20;28(9):1566-72. doi: 10.1200/JCO.2009.25.4680. Epub

2010 Feb 22.

Early detection of superficial squamous cell carcinoma in the head and neck

region and esophagus by narrow band imaging: a multicenter randomized controlled

trial.

Reason for exclusion: other topic: diagnostic instead of therapy

5. Journal of Clinical Oncology: Official Journal of the American Society of Clinical Oncology. DOI: 10.1200/JCO.2010.32.4103. Henke, M. et al. (2011).

Palifermin decreases severe oral mucositis of patients undergoing postoperative radiochemotherapy for head and neck cancer: a randomized, placebo-controlled trial.

Reason for exclusion: same study utilized as another article

**Table S1** Characteristics of the 139 included articles from 2020–2022

| Characteristics | Frequency | % |
| --- | --- | --- |
| *Year of publication* |  |  |
| 2020–2022 | 139 | 100.00 |
| *Continent of the first author* |  |  |
| Africa | 0 | 0.00 |
| Asia | 56 | 40.29 |
| Africa / Asia | 4 | 2.88 |
| Europe / Asia | 4 | 2.88 |
| Australia | 4 | 2.88 |
| Europe | 44 | 31.65 |
| North America | 18 | 12.95 |
| South America | 8 | 5.76 |
| Unclear | 4 | 2.88 |
| *Continent of the last author* |  |  |
| Africa | 0 | 0.00 |
| Asia | 52 | 37.41 |
| Africa / Asia | 2 | 1.44 |
| Europe / Asia | 1 | 0.72 |
| Australia | 4 | 2.88 |
| Europe | 38 | 27.34 |
| North America | 17 | 12.23 |
| South America | 7 | 5.04 |
| Unclear | 18 | 12.95 |
| *Journal type* |  |  |
| Oral oncology journal | 5 | 3.60 |
| Oncology journal | 73 | 52.52 |
| Other | 61 | 43.88 |
| *Journal name* |  |  |
| Supportive Care in Cancer | 9 | 6.47 |
| International Journal of Radiation Oncology  Biology Physics | 8 | 5.76 |
| Radiotherapy and Oncology | 6 | 4.32 |
| Journal of Clinical Oncology | 5 | 3.60 |
| Radiation Oncology | 5 | 3.60 |
| Others | 106 | 76.26 |
| *RCT type* |  |  |
| Parallel | 138 | 99.28 |
| Split-mouth | 0 | 0.00 |
| Other | 1 | 0.72 |
| *RCT aim* |  |  |
| Interventional | 139 | 100.00 |
| Other | 0 | 0.00 |
| *RCT arms* |  |  |
| 2 | 128 | 92.09 |
| 3 | 9 | 6.47 |
| 4 (and more) | 2 | 1.44 |
| *RCT blinding* |  |  |
| single-blind | 33 | 23.74 |
| double-blind and triple-blind | 40 | 28.78 |
| not reported / unclear | 24 | 17.27 |
| no blinding | 42 | 30.22 |
| *Main objective of the RCT* |  |  |
| treat cancer | 38 | 27.34 |
| treat or avoid the side effect of cancer treatment  and supportive treatment for cancer | 101 | 72.66 |
| *Number of centers of the study* |  |  |
| single center | 91 | 65.47 |
| multicenter | 35 | 25.18 |
| unclear | 13 | 9.35 |
| *Protocol registration?* |  |  |
| Yes | 112 | 80.58 |
| No | 27 | 19.42 |
| *Protocol registry name* |  |  |
| ClinicalTrials.gov | 61 | 43.88 |
| Clinical Trials Registry of India (CTRI) | 6 | 4.32 |
| Clinical Trials Registry of India (CTRI) | 5 | 3.60 |
| German Clinical Trials Register (DRKS) | 5 | 3.60 |
| Thai Clinical Trials Registry | 3 | 2.16 |
| Japan Registry of Clinical Trials (jRCT) | 3 | 2.16 |
| ISRCTN Registry | 3 | 2.16 |
| Brazilian Clinical Trials Registry (REBEC) | 3 | 2.16 |
| Iranian Registry of Clinical Trials (IRCT) | 3 | 2.16 |
| Australian New Zealand Clinical Trials  Registry (ACTRN) | 3 | 2.16 |
| Others | 17 | 12.23 |
| *Ethics committee reported* |  |  |
| Yes | 136 | 97.84 |
| No | 3 | 2.16 |
| *Conflict of interest statement reported* |  |  |
| Yes | 128 | 92.09 |
| No | 11 | 7.91 |
| *Statement on funding reported* |  |  |
| Yes | 123 | 88.49 |
| No | 16 | 11.51 |
| *Number of citations (Google Scholar)* |  |  |
| Median (IQR) | 5 (10) |  |
| *Impact factor (IF)* |  |  |
| Median (IQR) | 4.183 (4.028) |  |
| *H index of the first author* |  |  |
| Median (IQR) | 6 (14) |  |
| *H index of the last author* |  |  |
| Median (IQR) | 17 (22.5) |  |
| *Outcome measure used in the RCT* |  |  |
| Surrogate (non-PRO) | 30 | 21.58 |
| Patient-reported outcome (PRO) | 27 | 19.42 |
| Combination of both | 81 | 58.27 |
| Unclear | 1 | 0.72 |
| *Primary outcome reported?* |  |  |
| Yes | 104 | 74.82 |
| No | 35 | 25.18 |

**Table S2** Characteristics of the 45 included articles from 2010–2012

| Characteristics | Frequency | % |
| --- | --- | --- |
| *Year of publication* |  |  |
| 2010–2012 | 45 | 100.00 |
| *Continent of the first author* |  |  |
| Africa | 0 | 0.00 |
| Asia | 4 | 8.89 |
| Africa / Asia | 0 | 0.00 |
| Europe / Asia | 0 | 0.00 |
| Australia | 0 | 0.00 |
| Europe | 23 | 51.11 |
| North America | 10 | 22.22 |
| South America | 1 | 2.22 |
| Unclear | 7 | 15.56 |
| *Continent of the last author* |  |  |
| Africa | 0 | 0.00 |
| Asia | 5 | 11.11 |
| Africa / Asia | 0 | 0.00 |
| Europe / Asia | 0 | 0.00 |
| Australia | 0 | 0.00 |
| Europe | 21 | 46.67 |
| North America | 12 | 26.67 |
| South America | 1 | 2.22 |
| Unclear | 6 | 13.33 |
| *Journal type* |  |  |
| Oral oncology journal | 2 | 4.44 |
| Oncology journal | 30 | 66.67 |
| Other | 13 | 28.89 |
| *Journal name* |  |  |
| International Journal of Radiation Oncology  Biology Physics | 5 | 11.11 |
| Lancet Oncology | 5 | 11.11 |
| Radiotherapy and Oncology | 5 | 11.11 |
| Journal of Clinical Oncology | 3 | 6.67 |
| British Journal of Oral & Maxillofacial Surgery | 3 | 6.67 |
| Others | 24 | 53.33 |
| *RCT type* |  |  |
| Parallel | 45 | 100.00 |
| Split-mouth | 0 | 0.00 |
| Other | 0 | 0.00 |
| *RCT aim* |  |  |
| Interventional | 45 | 100.00 |
| Other | 0 | 0.00 |
| *RCT arms* |  |  |
| 2 | 39 | 86.67 |
| 3 | 5 | 11.11 |
| 4 (and more) | 1 | 2.22 |
| *RCT blinding* |  |  |
| single-blind | 6 | 13.33 |
| double-blind and triple-blind | 13 | 28.89 |
| not reported / unclear | 16 | 35.56 |
| no blinding | 10 | 22.22 |
| *Main objective of the RCT* |  |  |
| treat cancer | 19 | 42.22 |
| treat or avoid the side effect of cancer treatment  and supportive treatment for cancer | 26 | 57.78 |
| *Number of centers of the study* |  |  |
| single center | 11 | 24.44 |
| multicenter | 18 | 40.00 |
| unclear | 16 | 35.56 |
| *Protocol registration?* |  |  |
| Yes | 15 | 33.33 |
| No | 30 | 66.67 |
| *Protocol registry name* |  |  |
| ClinicalTrials.gov | 10 | 66.67 |
| Netherlands Trial Registry (NTR) | 1 | 6.67 |
| National Research Register | 1 | 6.67 |
| Clinical Trials Registry of India (CTRI) | 1 | 6.67 |
| ISRCTNregistry | 1 | 6.67 |
| CKTO | 1 | 6.67 |
| *Ethics committee reported* |  |  |
| Yes | 34 | 75.56 |
| No | 11 | 24.44 |
| *Conflict of interest statement reported* |  |  |
| Yes | 27 | 60.00 |
| No | 18 | 40.00 |
| *Statement on funding reported* |  |  |
| Yes | 26 | 57.78 |
| No | 19 | 42.22 |
| *Number of citations (Google Scholar)* |  |  |
| Median (IQR) | 91 (137.5) |  |
| *Impact factor (IF)* |  |  |
| Median (IQR) | 6.901 (6.5845) |  |
| *H index of the first author* |  |  |
| Median (IQR) | 22 (37) |  |
| *H index of the last author* |  |  |
| Median (IQR) | 40 (35) |  |
| *Outcome measure used in the RCT* |  |  |
| Surrogate (non-PRO) | 12 | 26.67 |
| Patient-reported outcome (PRO) | 4 | 8.89 |
| Combination of both | 29 | 64.44 |
| *Primary outcome reported?* |  |  |
| Yes | 33 | 73.33 |
| No | 12 | 26.67 |

**Table S3** Characteristics of the 20 included articles from 2000–2002

| Characteristics | Frequency | % |
| --- | --- | --- |
| *Year of publication* |  |  |
| 2000–2002 | 20 | 100.00 |
| *Continent of the first author* |  |  |
| Africa | 0 | 0.00 |
| Asia | 3 | 15.00 |
| Africa / Asia | 0 | 0.00 |
| Europe / Asia | 0 | 0.00 |
| Australia | 0 | 0.00 |
| Europe | 8 | 40.00 |
| North America | 7 | 35.00 |
| South America | 0 | 0.00 |
| Unclear | 2 | 10.00 |
| *Continent of the last author* |  |  |
| Africa | 0 | 0.00 |
| Asia | 1 | 5.00 |
| Africa / Asia | 0 | 0.00 |
| Europe / Asia | 0 | 0.00 |
| Australia | 2 | 10.00 |
| Europe | 11 | 55.00 |
| North America | 2 | 10.00 |
| South America | 0 | 0.00 |
| Unclear | 4 | 20.00 |
| *Journal type* |  |  |
| Oral oncology journal | 1 | 5.00 |
| Oncology journal | 16 | 80.00 |
| Other | 3 | 15.00 |
| *Journal name* |  |  |
| International Journal of Radiation  Oncology Biology Physics | 3 | 15.00 |
| Cancer | 2 | 10.00 |
| European Journal of Cancer (EJC) | 2 | 10.00 |
| Acta Oncologica | 2 | 10.00 |
| Radiotherapy and Oncology | 2 | 10.00 |
| Others | 9 | 45.00 |
| *RCT type* |  |  |
| Parallel | 20 | 100.00 |
| Split-mouth | 0 | 0.00 |
| Other | 0 | 0.00 |
| *RCT aim* |  |  |
| Interventional | 20 | 100.00 |
| Other | 0 | 0.00 |
| *RCT arms* |  |  |
| 2 | 20 | 100.00 |
| *RCT blinding* |  |  |
|  | 0 | 0.00 |
| double-blind and triple-blind | 9 | 45 |
| not reported / unclear | 10 | 50 |
| no blinding | 1 | 5 |
| *Main objective of the RCT* |  |  |
| treat cancer | 11 | 55 |
| treat or avoid the side effect of cancer  treatment and supportive treatment for  cancer | 9 | 45 |
| *Number of centers of the study* |  |  |
| single center | 9 | 45 |
| multicenter | 9 | 45 |
| unclear | 2 | 10 |
| *Protocol registration?* |  |  |
| Yes | 0 | 0.00 |
| No | 20 | 100.00 |
| *Ethics committee reported* |  |  |
| Yes | 9 | 45.00 |
| No | 11 | 55.00 |
| *Conflict of interest statement reported* |  |  |
| Yes | 0 | 0.00 |
| No | 20 | 100.00 |
| *Statement on funding reported* |  |  |
| Yes | 6 | 30.00 |
| No | 14 | 70.00 |
| *Number of citations (Google Scholar)* |  |  |
| Median (IQR) | 73.5 (144.5) |  |
| *Impact factor (IF)* |  |  |
| Median (IQR) | 6.901 (3.9075) |  |
| *H index of the first author* |  |  |
| Median (IQR) | 20.5 (38.25) |  |
| *H index of the last author* |  |  |
| Median (IQR) | 25.5 (37.5) |  |
| *Outcome measure used in the RCT* |  |  |
| Surrogate (non-PRO) | 8 | 40.00 |
| Patient-reported outcome (PRO) | 1 | 5.00 |
| Combination of both | 11 | 55.00 |
| *Primary outcome reported?* |  |  |
| Yes | 13 | 65 |
| No | 7 | 35 |

**Table S4**  The 20 most prevalent non-patient reported outcomes (non-PROs) in the 139 included articles from 2020–2022

| Outcome | N | (%) |
| --- | --- | --- |
| 1. Overall survival | 43 | 30.94 |
| 1. Progression-free survival | 25 | 17.99 |
| 1. Adverse events, adverse effects, serious adverse events | 24 | 17.27 |
| 1. Objective response rate, overall response rate, response rate, tumor response | 15 | 10.79 |
| 1. Oral mucositis, mucositis, severe oral mucositis, radiation induced oral mucositis | 14 | 10.07 |
| 1. Toxicity, acute and late toxicity | 14 | 10.07 |
| 1. Disease specific survival, disease free survival | 12 | 8.63 |
| 1. Locoregional control | 11 | 7.91 |
| 1. Blood loss, blood pressure, blood tests | 10 | 7.19 |
| 1. Amount of saliva, sticky saliva, saliva production, salivary flow | 9 | 6.47 |
| 1. Safety of treatments | 6 | 4.32 |
| 1. Morbidity | 5 | 3.60 |
| 1. Analgesia, opiod use | 5 | 3.60 |
| 1. Hospitalisation, hospital stay, days in the hospital | 4 | 2.88 |
| 1. Oral health | 4 | 2.88 |
| 1. Dermatitis | 4 | 2.88 |
| 1. Tumor recurrences | 4 | 2.88 |
| 1. Xerostomia | 3 | 2.16 |
| 1. Dysphagia | 3 | 2.16 |
| 1. Death | 3 | 2.16 |

**Table S5** The 20 most prevalent non-patient reported outcomes (non-PROs) in the 45 included articles from 2010–2012

| Outcome | N | (%) |
| --- | --- | --- |
| 1. Overall survival | 21 | 46.67 |
| 1. Objective response rate, overall response rate, response rate, tumor response | 10 | 22.22 |
| 1. Adverse events, adverse effects, serious adverse events | 10 | 22.22 |
| 1. Progression-free survival, event free survival, relapse free survival | 9 | 20.00 |
| 1. Toxicity, acute and late toxicity | 8 | 17.78 |
| 1. Locoregional control | 8 | 17.78 |
| 1. Oral mucositis, Mucositis, severe oral mucositis, radiation induced oral mucositis | 6 | 13.33 |
| 1. Xerostomia | 5 | 11.11 |
| 1. Disease specific survival, disease free survival | 5 | 11.11 |
| 1. Distant metastasis, metastases | 5 | 11.11 |
| 1. Dysphagia | 5 | 11.11 |
| 1. Analgesia, opiod use | 3 | 6.67 |
| 1. Morbidity | 3 | 6.67 |
| 1. Edema | 3 | 6.67 |
| 1. Tracheostomy feeding-tube dependence; need for tube feeding, feeding tube | 3 | 6.67 |
| 1. Amount of saliva, sticky saliva, saliva production, salivary flow | 2 | 4.44 |
| 1. Safety of treatments | 2 | 4.44 |
| 1. Healing time | 2 | 4.44 |
| 1. Postoperative infection, wound infection | 2 | 4.44 |
| 1. Hospitalisation, hospital stay, days in the hospital | 2 | 4.44 |

**Table S6** The 20 most prevalent non-patient reported outcomes (non-PROs) in the 20 included articles from 2000–2002

| Outcome | N | (%) |
| --- | --- | --- |
| 1. Overall survival | 8 | 40 |
| 1. Toxicity, acute and late toxicity | 8 | 40 |
| 1. Oral mucositis, Mucositis, severe oral mucositis, radiation induced oral mucositis | 7 | 35 |
| 1. Objective response rate, overall response rate, response rate, tumor response | 4 | 20 |
| 1. Disease specific survival, disease free survival | 4 | 20 |
| 1. Xerostomia | 4 | 20 |
| 1. Distant metastasis, metastases | 4 | 20 |
| 1. Amount of saliva, sticky saliva, saliva production, salivary flow | 3 | 15 |
| 1. Locoregional control | 3 | 15 |
| 1. Morbidity | 3 | 15 |
| 1. Death | 2 | 10 |
| 1. Oral health | 2 | 10 |
| 1. Compliance with treatment | 2 | 10 |
| 1. Tumor recurrences | 2 | 10 |
| 1. Ulceration | 2 | 10 |
| 1. Erythema | 2 | 10 |
| 1. Adverse events, adverse effects, serious adverse events | 1 | 5 |
| 1. Blood loss, blood pressure, blood tests | 1 | 5 |
| 1. Dysphagia | 1 | 5 |
| 1. Progression-free survival | 1 | 5 |

**Table S7** The 20 most prevalent patient-reported outcomes (PROs) in the 139 included articles from 2020–2022

| Outcome | N | (%) |
| --- | --- | --- |
| 1. Pain | 47 | 33.81 |
| 1. Quality of life | 44 | 31.65 |
| 1. Adverse events, adverse effects, serious adverse events | 17 | 12.23 |
| 1. Swallowing, swallowing difficulties, swallowing problems | 13 | 9.35 |
| 1. Xerostomia, dry mouth | 13 | 9.35 |
| 1. Weight, weight loss, weight changes, body weight (BMI) | 12 | 8.63 |
| 1. Nausea | 11 | 7.91 |
| 1. Toxicity | 11 | 7.91 |
| 1. Depression | 9 | 6.47 |
| 1. Amount of saliva, sticky saliva, saliva production, salivary flow | 8 | 5.76 |
| 1. Patients´ satisfaction | 8 | 5.76 |
| 1. Fatigue | 6 | 4.32 |
| 1. Compliance, treatment compliance | 6 | 4.32 |
| 1. Patients´ adherence to treatment | 6 | 4.32 |
| 1. Vomiting | 6 | 4.32 |
| 1. Taste, taste loss, taste changes | 5 | 3.60 |
| 1. Mouth opening | 4 | 2.88 |
| 1. Social eating, social functioning | 4 | 2.88 |
| 1. Appetite loss | 3 | 2.16 |
| 1. Symptom burden | 3 | 2.16 |

**Table S8** The 20 most prevalent patient-reported outcomes (PROs) in the 45 included articles from 2010–2012

| Outcome | N | (%) |
| --- | --- | --- |
| 1. Pain | 12 | 26.67 |
| 1. Quality of life | 11 | 24.44 |
| 1. Weight, weight loss, weight changes, body weight (BMI) | 8 | 17.78 |
| 1. Xerostomia, dry mouth | 7 | 15.56 |
| 1. Adverse events, adverse effects, serious adverse events | 4 | 8.89 |
| 1. Nausea | 3 | 6.67 |
| 1. Swallowing, swallowing difficulties, swallowing problems | 3 | 6.67 |
| 1. Taste, taste loss, taste changes | 3 | 6.67 |
| 1. Medication, analgesics use | 3 | 6.67 |
| 1. Vomiting | 2 | 4.44 |
| 1. Toxicity | 2 | 4.44 |
| 1. Fatigue | 2 | 4.44 |
| 1. Social eating, social functioning, eating in public, eating | 2 | 4.44 |
| 1. Mouth opening | 2 | 4.44 |
| 1. Hospitalisation, hospital stay, days in the hospital | 2 | 4.44 |
| 1. Amount of saliva, sticky saliva, saliva production, salivary flow | 1 | 2.22 |
| 1. Compliance, treatment compliance | 1 | 2.22 |
| 1. Constipation | 1 | 2.22 |
| 1. Depression | 1 | 2.22 |
| 1. Smoking and alcohol habits | 1 | 2.22 |

**Table S9** The 20 most prevalent patient-reported outcomes (PROs) in the 20 included articles from 2000–2002

| Outcome | N | (%) |
| --- | --- | --- |
| 1. Pain | 7 | 35 |
| 1. Vomiting | 4 | 20 |
| 1. Adverse events, adverse effects, serious adverse events | 3 | 15 |
| 1. Weight, weight loss, weight changes, body weight (BMI) | 3 | 15 |
| 1. Nausea | 3 | 15 |
| 1. Xerostomia, dry mouth | 3 | 15 |
| 1. Quality of life | 2 | 10 |
| 1. Swallowing, swallowing difficulties, swallowing problems | 2 | 10 |
| 1. Taste loss, taste changes | 2 | 10 |
| 1. Speaking problems | 2 | 10 |
| 1. Depression | 1 | 5 |
| 1. Sleep, sleep problems | 1 | 5 |
| 1. Constipation | 1 | 5 |
| 1. Performance status | 1 | 5 |
| 1. Food intake | 1 | 5 |
| 1. Sweating | 1 | 5 |
| 1. Dizziness | 1 | 5 |
| 1. Urinary frequency | 1 | 5 |
| 1. Need for oral comfort aids | 1 | 5 |
| 1. Itching | 1 | 5 |

**Table S10**  Types of instruments utilized for non-patient reported outcomes (non-PROs) of the 139 included articles from 2020–2022

| Outcome | Measured by: |
| --- | --- |
| 1. Overall survival | Date of diagnosis until the date of death or the last follow-up, Kaplan-Meier method |
| 1. Progression-free survival | Kaplan-Meier Method |
| 1. Adverse events, adverse effects, serious adverse events | CTACE version 3.0/ 4.0/ 4.02/ 5.0 |
| 1. Objective response rate, overall response rate, response rate, tumor response | Time to objective response, overall response rate (ORR) |
| 1. Oral mucositis, mucositis, severe oral mucositis, radiation induced oral mucositis | CTCAE V4.0 and the Oral Mucosal Assessment Scale ulcer score, Mucositis grade by WHO |
| 1. Toxicity, acute and late toxicity | CTC 3.0, EORTC/RTOG scale, European Organization for Research and Treatment of Cancer (EORTC), Radiation Oncology Group (RTOG) |
| 1. Disease specific survival, disease free survival | Time; disease-free actuarial survival rates |
| 1. Locoregional control | Kaplan-Meier Method |
| 1. Blood loss, blood pressure, blood tests | Intraoperative blood loss, blood tests |
| 1. Amount of saliva, sticky saliva, saliva production, salivary flow | UWSFR (unstimulated whole salivary flow rates); SSFR (stimulated salivary flow rates) |
| 1. Safety of treatments | Obtained from electronic patient records |
| 1. Morbidity | Early and late treatment-related morbidity |
| 1. Analgesia, opiod use | Duration and dose of use of opiate analgesia |
| 1. Hospitalisation, hospital stay, days in the hospital | Time |
| 1. Oral health | Oral health status, measured by a nurse |
| 1. Dermatitis | Grading based on The National Cancer Institute Common Toxicity Criteria for Adverse Events |
| 1. Tumor recurrences | Local and regional tumor recurrences |
| 1. Xerostomia | Clinical symptoms of xerostomia by RIXVAS (radiation-induced xerostomia visual analogue scale): the objective grade by two separate observers |
| 1. Dysphagia | MDADI (MD Anderson Dysphagia Inventory) |
| 1. Death | Death rate |

**Table S11**  Types of instruments utilized for non-patient reported outcomes (non-PROs) of the 45 included articles from 2010–2012

| Outcome | Measured by: |
| --- | --- |
| 1. Overall survival | Date of diagnosis until the date of death or the last follow-up, Kaplan-Meier method: relapse-free survival |
| 1. Objective response rate, overall response rate, response rate, tumor response | Time to objective response, overall response rate (ORR) |
| 1. Adverse events, adverse effects, serious adverse events | Common Terminology Criteria for Adverse Events version 3.0 (CTCAE v3.0) |
| 1. Progression-free survival, event free survival, relapse free survival | Kaplan-Meier Method |
| 1. Toxicity, acute and late toxicity | Blood tests |
| 1. Locoregional control | Locoregional control rates, Kaplan-Meier Method |
| 1. Oral mucositis, Mucositis, severe oral mucositis, radiation induced oral mucositis | WHO grading for mucositis |
| 1. Xerostomia | Clinical symptoms of xerostomia by RIXVAS (radiation-induced xerostomia visual analogue scale), the objective grade by two separate observers, RTOG |
| 1. Disease specific survival, disease free survival | Time; disease-free actuarial survival rates |
| 1. Distant metastasis, metastases | Regional metastasis, defined as nodal positive either identified from elective neck dissection at the time of surgery or from post-surgery follow-up, or distant metastasis |
| 1. Dysphagia | MD Anderson Dysphagia Inventory (MDADI) questionnaire |
| 1. Analgesia, opiod use | Duration and dose of use of opiate analgesia |
| 1. Morbidity | Early and late treatment-related morbidity |
| 1. Edema | Objective signs |
| 1. Tracheostomy feeding-tube dependence; need for tube feeding, feeding tube | Objective signs |
| 1. Amount of saliva, sticky saliva, saliva production, salivary flow | UWSFR (unstimulated whole salivary flow rates); SSFR (stimulated salivary flow rates); Salivary gland scintigraphy, saliva production |
| 1. Safety of treatments | Obtained from electronic patient records |
| 1. Healing time | Time |
| 1. Postoperative infection, wound infection | Objective signs |
| 1. Hospitalisation, hospital stay, days in the hospital | Time |

**Table S12**  Types of instruments utilized for non-patient reported outcomes (non-PROs) of the 20 included articles from 2000–2002

| Outcome | Measured by: |
| --- | --- |
| 1. Overall survival | Overall 5-year survival rate (by Kaplan-Meier method) |
| 1. Toxicity, acute and late toxicity | EORTC/RTOG scale, European Organization for Research and Treatment of Cancer (EORTC), Radiation Oncology Group (RTOG) |
| 1. Oral mucositis, Mucositis, severe oral mucositis, radiation induced oral mucositis | Mucositis grade by WHO |
| 1. Objective response rate, overall response rate, response rate, tumor response | Time to objective response, overall response rate (ORR) |
| 1. Disease specific survival, disease free survival | Time; disease-free actuarial survival rates |
| 1. Xerostomia | The objective grade by two separate observers |
| 1. Distant metastasis, metastases | Regional metastasis, defined as nodal positive either identified from elective neck dissection at the time of surgery or from postsurgery follow-up, or distant metastasis |
| 1. Amount of saliva, sticky saliva, saliva production, salivary flow | Salivary gland scintigraphy; saliva production |
| 1. Locoregional control | Locoregional control rates, Kaplan-Meier Method |
| 1. Morbidity | Early and late treatment-related morbidity |
| 1. Death | Death rate and status of death |
| 1. Oral health | Oral health status, measured by a nurse |
| 1. Compliance with treatment | Treatment brakes |
| 1. Tumor recurrences | Local and regional tumor recurrences |
| 1. Ulceration | EORTC/RTOG acute skin reaction scoring system |
| 1. Erythema | EORTC/RTOG acute skin reaction scoring system |
| 1. Adverse events, adverse effects, serious adverse events | Common Terminology Criteria for Adverse Events version 3.0 (CTCAE v3.0) |
| 1. Blood loss, blood pressure, blood tests | Blood loss during surgery |
| 1. Dysphagia | WHO scale and RTOG criteria |
| 1. Progression-free survival | Kaplan-Meier Method |

**Table S13**  Types of instruments utilized for patient reported outcomes (PROs) of the 139 included articles from 2020–2022

| Outcome | Measured by: |
| --- | --- |
| 1. Pain | VAS |
| 1. Quality of life | QLQ-C30, Quality of Life Questionnaire includes 30 items grouped into five quality of life categories: physical, social, emotional, cognitive and role performance; Core Module (QLQ-C30) and Head and Neck Module (QLQ-H&N35) |
| 1. Adverse events, adverse effects, serious adverse events | Questionnaire |
| 1. Swallowing, swallowing difficulties, swallowing problems | FOIS, PSS-H&N |
| 1. Xerostomia, dry mouth | XQ (Xerostomia Questionnaire), TESS (Treatment-Emergent Symptom Scale), VAS |
| 1. Weight, weight loss, weight changes, body weight (BMI) | Questionnaire, weight measurement |
| 1. Nausea | Questionnaire |
| 1. Toxicity | European Organization for Research and Treatment of Cancer (EORTC), questionnaire |
| 1. Depression | Assessed with PROMIS, (by EQ-5D-3L and EORTC QLU-C10D) |
| 1. Amount of saliva, sticky saliva, saliva production, salivary flow | UWSFR (unstimulated whole salivary flow rates); SSFR (stimulated salivary flow rates) |
| 1. Patients´ satisfaction | Questionaire; the Patient Concerns Inventory (PCI); preoperative and postoperative questionnaires |
| 1. Fatigue | BFI (Brief Fatigue Inventory), global fatigue score |
| 1. Compliance, treatment compliance | Questionnaire |
| 1. Patients´ adherence to treatment | Participants rated the extent to which they followed their treatment plan and followed their doctor’s instructions; using MUSE (the Medication and Use Self-Efficacy scale) |
| 1. Vomiting | Questionnaire |
| 1. Taste, taste loss, taste changes | Questionnaire |
| 1. Mouth opening | Maximum mouth opening |
| 1. Social eating, social functioning | EORTC QLQ-H&N35 |
| 1. Appetite loss | Questionnaire |
| 1. Symptom burden | H&N35; ESAS (the Edmonton Symptom Assessment System) |

**Table S14**  Types of instruments utilized for patient reported outcomes (PROs) of the 45 included articles from 2010–2012

| Outcome | Measured by: |
| --- | --- |
| 1. Pain | VAS |
| 1. Quality of life | (EORTC QLQ-C30), (EORTC QLQ-HN35, EORTC QLQ-LC13), (EORTC QLQ-PATSAT) |
| 1. Weight, weight loss, weight changes, body weight (BMI) | Questionnaire, weight measurement |
| 1. Xerostomia, dry mouth | XQ (Xerostomia Questionnaire) |
| 1. Adverse events, adverse effects, serious adverse events | Questionnaire |
| 1. Nausea | Questionnaire |
| 1. Swallowing, swallowing difficulties, swallowing problems | Swallowing function (by FOIS and PSS-H&N) |
| 1. Taste, taste loss, taste changes | Questionnaire |
| 1. Medication, analgesics use | Questionnaire |
| 1. Vomiting | Questionnaire, medication taken |
| 1. Toxicity | European Organization for Research and Treatment of Cancer (EORTC), questionnaire |
| 1. Fatigue | Questionnaire |
| 1. Social eating, social functioning, eating in public, eating | Questionnaire |
| 1. Mouth opening | Questionnaire |
| 1. Hospitalisation, hospital stay, days in the hospital | Time, questionnaire |
| 1. Amount of saliva, sticky saliva, saliva production, salivary flow | UWSFR (unstimulated whole salivary flow rates); SSFR (stimulated salivary flow rates) |
| 1. Compliance, treatment compliance | Questionnaire |
| 1. Constipation | Questionnaire |
| 1. Depression | Questionnaire, the Hospital Anxiety and Depression Scale (HADS) |
| 1. Smoking and alcohol habits | Questionnaire |

**Table S15** Types of instruments utilized for patient reported outcomes (PROs) of the 20 included articles from 2000–2002

| Outcome | Measured by: |
| --- | --- |
| 1. Pain | VAS and the McGill Pain Questionnaire |
| 1. Vomiting | Questionnaire |
| 1. Adverse events, adverse effects, serious adverse events | Questionnaire |
| 1. Weight, weight loss, weight changes, body weight (BMI) | Questionnaire, weight measurement |
| 1. Nausea | Questionnaire |
| 1. Xerostomia, dry mouth | VAS |
| 1. Quality of life | Questionnaire; the general cancer-specific European Organization for Research and Treatment of Cancer (EORTC) Quality of Life-score 30 (QLQ-C30) instrument |
| 1. Swallowing, swallowing difficulties, swallowing problems | Questionaire: PBQ (patient benefit questionnaire) |
| 1. Taste loss, taste changes | Questionnaire |
| 1. Speaking problems | Questionnaire |
| 1. Depression | Questionnaire; Beck's Depression Inventory |
| 1. Sleep, sleep problems | Questionnaire |
| 1. Constipation | Questionnaire |
| 1. Performance status | Questionnnaire |
| 1. Food intake | Questionnairee, by the WHO grading system |
| 1. Sweating | Questionnaire |
| 1. Dizziness | Questionnaire |
| 1. Urinary frequency | Questionnaire |
| 1. Need for oral comfort aids | Questionnaire |
| 1. Itching | Questionnaire |

**Table S16** Outcome measure used in the RCT for each period

| ****Period (Studies)**** | ****non-PRO (%)**** | ****PRO****  ****(%)**** | ****Combination (%)**** | ****Unclear (%)**** |
| --- | --- | --- | --- | --- |
| 2000 – 2002 ; (20) | 8 (40) | 1 (5) | 11 (55) | 0 (0) |
| 2010 – 2012 ; (45) | 12 (26.67) | 4 (8.89) | 29 (64.44) | 0 (0) |
| 2020 – 2022 ; (139) | 30 (21.58) | 27 (19.42) | 81 (58.27) | 1 (0.72) |
